# Picornaviruses: A View from 3A

**DOI:** 10.3390/v13030456

**Published:** 2021-03-11

**Authors:** Terry Jackson, Graham J. Belsham

**Affiliations:** 1 The Pirbright Institute, Pirbright, Woking, Surrey GU24 0NF, UK; terry.jackson@pirbright.ac.uk; 2 Department of Veterinary and Animal Sciences, University of Copenhagen, 1870 Frederiksberg, Denmark

**Keywords:** virus replication, replication organelles, protein interactions, membrane interactions, protein trafficking, lipid droplets, virus host-range, phosphatidylinositol 4-kinase, RNA replication

## Abstract

Picornaviruses are comprised of a positive-sense RNA genome surrounded by a protein shell (or capsid). They are ubiquitous in vertebrates and cause a wide range of important human and animal diseases. The genome encodes a single large polyprotein that is processed to structural (capsid) and non-structural proteins. The non-structural proteins have key functions within the viral replication complex. Some, such as 3D^pol^ (the RNA dependent RNA polymerase) have conserved functions and participate directly in replicating the viral genome, whereas others, such as 3A, have accessory roles. The 3A proteins are highly divergent across the *Picornaviridae* and have specific roles both within and outside of the replication complex, which differ between the different genera. These roles include subverting host proteins to generate replication organelles and inhibition of cellular functions (such as protein secretion) to influence virus replication efficiency and the host response to infection. In addition, 3A proteins are associated with the determination of host range. However, recent observations have challenged some of the roles assigned to 3A and suggest that other viral proteins may carry them out. In this review, we revisit the roles of 3A in the picornavirus life cycle. The 3AB precursor and mature 3A have distinct functions during viral replication and, therefore, we have also included discussion of some of the roles assigned to 3AB.

## 1. Picornaviruses

Picornaviruses are ubiquitous in nature and have been identified in all vertebrate classes [1]. They are responsible for many diseases of medical importance, such as poliomyelitis (caused by poliovirus; PV), hand-foot-and mouth disease (caused by coxsackievirus (CV) A16; CVA9, and enterovirus A71; EV-A71) and the common cold (caused by human rhinoviruses; RV) [2]. They are also responsible for some important veterinary diseases, notably foot-and-mouth disease (caused by foot-and-mouth disease virus; FMDV) [3,4].

Picornaviruses are small and non-enveloped; they have positive-sense RNA genomes. Over the last two decades, many new picornaviruses have been identified and currently the picornavirus family (the *Picornaviridae*) is composed of 47 genera with over 100 different species [5]. As new picornaviruses are characterized, it has become clear that they show considerable diversity in their genome organization and proteins (Table 1) [6,7,8,9]. The 3A proteins of picornaviruses are some of the most divergent across the different genera. They have important roles in forming both the viral replication complex (RC) and replication organelles (RO), and are also involved in pathogenesis and determining host range. However, most of the current knowledge of picornavirus biology comes from studies on mammalian picornaviruses from the *Enterovirus, Cardiovirus, Aphthovirus* and *Kobuvirus* genera, and therefore the 3A proteins of these viruses are the main focus of this review. The 3A proteins are present within infected cells, not only in their mature form but also as part of larger 3A-containing precursor proteins, notably 3AB. It has been known for many years that 3A and 3AB have distinct roles in the picornavirus replication cycle and, therefore, a discussion of the main roles of 3AB in picornavirus replication is also included. 

## 2. Key Features of Picornavirus Genomes

A typical picornavirus genome (ca. 7.5–8.5kb) includes a single large open reading frame (ORF) flanked by 5′- and 3′-untranslated regions (UTRs) (Table 1). As a consequence of the RNA replication mechanism, the 5′-terminus of the genomic RNA is covalently linked to a small virus-encoded peptide, VPg (viral protein genome-linked), which is encoded by the 3B region of the genome [10,11,12,13,14]. The 5′-UTR varies in length (typically between about 650 and 1300 nucleotides (nt)) across different picornaviruses and includes several structural elements that are critical for virus replication. These include the IRES (internal ribosome entry site), which directs cap-independent initiation of protein synthesis, and also genus-specific elements such as the cloverleaf (CL: enteroviruses) and the S fragment (aphthoviruses), which are required for viral RNA (vRNA) replication [6,15,16,17,18,19]. The 3′-UTR is usually relatively short (< 100 nt) and followed by a poly(A) tail. The poly(A) tail, and the unique sequences and structures in the 3′-UTR are required for negative-strand vRNA synthesis [20,21,22,23,24,25]. In addition, picornavirus genomes also contain other RNA elements that are required for vRNA replication such as the position-independent, internal *cis*-acting replication element (*cre*), which serves as the template for uridylylation of VPg (see below) [26,27,28,29,30]. For many picornaviruses (e.g., enteroviruses) the region of the genome that includes the ORF is divided into three main regions encoding the precursor proteins P1, P2 and P3; the P1 precursor is cleaved to the capsid proteins, while the P2 and P3 precursors are processed to the non-structural proteins (nsps). However, for some other picornaviruses (e.g., aphthoviruses and cardioviruses) the capsid precursor is P1-2A; thus, to cover both forms, the terms P1 and P1-(2A) are used here. For some picornaviruses, such as the aphthoviruses, cardioviruses and kobuviruses, the genome also encodes a Leader (L) protein at the N-terminus of the polyprotein sequence (Table 1).

## 3. Key Features of the Picornavirus Replication-Cycle

Picornavirus entry into cells culminates in the delivery of the infectious viral genome into the cytosol, where vRNA replication occurs [31,32,33]). During replication, RNA structures within the UTRs and coding regions of the genome cooperate to regulate initiation of viral protein synthesis and the sequential synthesis of negative- and positive-strand vRNA [20,21,25]. Initially, the genomic vRNA functions as a mRNA and translation initiation is directed by the IRES using a cap-independent mechanism [6]. For PV and FMDV, the CL and structures within the 3′-UTR, respectively, modulate IRES function [34,35,36,37]. The full-length viral polyprotein is never observed as it is rapidly processed, both co- and post-translationally (in *cis* and in *trans*), by virus-encoded proteases. These are: 2A^pro^, 3C^pro^ and 3CD^pro^ (enteroviruses); L^pro^ and 3C^pro^ (aphthoviruses); 3C^pro^ and 3CD^pro^ (cardioviruses and kobuviruses). Processing generates the L protein (for aphthoviruses, cardioviruses and kobuviruses) and the primary precursor polyproteins, P1 or P1-(2A) (dependent on the location of the primary cleavage at either the N-terminus or C-terminus of 2A), P2 and P3 [38,39,40,41]. The L protein (L^pro^) of FMDV is a papain-like protease and cleaves at the junction between its own C-terminus and the N-terminal Gly residue of P1-2A [42], whereas the L proteins of cardioviruses and kobuviruses are not proteases and are cleaved from P1-2A (cardioviruses) or P1 (kobuviruses) by 3C*^pro^* /3CD*^pro^* [43,44]. Further processing of the P1/P1-(2A), P2 and P3 precursors generates the mature viral proteins. The P1/P1-(2A) precursor is cleaved to make the capsid proteins (VP0, VP3 and VP1) plus, where applicable, 2A, while P2 and P3 precursors are processed to the nsps (P2: 2A (except where it is part of P1-2A), 2B and 2C; P3: 3A, 3B, 3C^pro^ and 3D^pol^). Processing also generates a number of processing intermediates (e.g., 2BC, 3AB and 3CD), which have distinct functions during virus replication [45]. This is important since the processing can have a major effect on the biological activities of the proteins, e.g., 3D^pol^ is the functional RNA polymerase whereas 3CD lacks this activity but does have protease activity (3CD^pro^). The P2 and P3 proteins (including some processing intermediates) orchestrate vRNA replication and the biochemical and morphological changes that occur inside infected cells. The 2A proteins differ significantly in their properties and some picornaviruses encode more than one 2A protein, these can have diverse functions (Table 1). For example, the enterovirus 2A is a protease (2A^pro^) that cleaves the P1/2A junction thus releasing the P1 capsid precursor but in other picornaviruses, e.g., kobuviruses, the 2A is not a protease and P1 is separated from 2A by 3CD^pro^ [46]. In the aphthoviruses (e.g., FMDV) the 2A peptide, which is only 18 residues long, induces a “ribosomal skip” within a NPG/P motif at the 2A/2B junction, such that the polypeptide bond at the G/P junction is not made [47]. The FMDV 2A peptide is closely related to the C-terminus of cardiovirus (and some other) 2A proteins that are believed to employ the same mechanism to separate the capsid precursor P1-2A from the P2 proteins [48]. 

Early during infection, picornaviruses shut down host-cell cap-dependent translation, which diverts translation away from cellular mRNAs to translation of the vRNA [49]. In addition to being translated, the input vRNA also serves as a template for the synthesis of negative-sense copies of the vRNA. It is widely believed that the process of RNA replication results in the formation of a double-stranded (ds)-RNA product (the replicative form) and then proceeds via the formation of a highly branched structure, termed the replicative intermediate (reviewed in [50]). However, it is possible that the presence of dsRNA in extracted vRNA is essentially a consequence of the presence of complementary positive and negative sense RNAs. It is not clear how the strands of a dsRNA molecule can be separated since single stranded forms of the RNA are required for both translation and RNA replication. It is interesting to note that the Qβ bacteriophage, with a small positive-sense RNA genome and an RNA dependent RNA polymerase, has been shown to have a mechanism for separating the positive and negative strands of RNA during synthesis (reviewed in [51]). This strand separation mechanism, which limits the length of the dsRNA to only 9 base pairs (bp), requires interaction between the RNA polymerase and the host proteins EF-Tu, EF-Ts and the ribosomal S1 protein. It seems like an elegant mechanism to keep the vRNA molecules separate. The production of dsRNA acts as a stimulator of anti-viral responses (see below), which could be counterproductive for the virus. Whatever the precise process of their synthesis, the newly formed negative-sense copies are then used as the template to synthesize more positive-sense vRNA [52], which can either be used to produce more viral proteins or can be packaged into progeny virions. Synthesis of both negative- and positive-sense vRNA is catalyzed by 3D^pol^, the viral RNA dependent RNA polymerase [53], and takes place within the viral RC, which forms in association with cellular proteins and membranes. In addition, 3D^pol^ also catalyzes VPg uridylylation (i.e., UMP linkage to the hydroxyl group of Tyr 3 within VPg) to generate VPg-pUpU-OH, which serves as the primer for both positive and negative strand vRNA synthesis [10,11,13,14]. Most picornaviruses encode a single copy of VPg (3B) (Table 1); however, some have two copies [54], whereas FMDV is unusual and possesses three non-identical copies of VPg [55,56] that all appear to be used for vRNA replication [29,57]. Positive-sense vRNA is packaged to form mature virions in a process that is believed to be dependent on packaging signals within the vRNA sequence, and also certain viral nsps and host factors [58,59,60,61,62]. For most picornaviruses, the capsid precursor undergoes post-translational N-terminal myristoylation, which is required for virion/capsid assembly [63,64,65] and also for VP4-induced membrane translocation of the vRNA during cell entry [66,67]. However, myristoylation of P1/P1-2A is not conserved among all picornaviruses and is absent in hepatoviruses and parechoviruses [68,69]. Encapsidation of the vRNA is usually accompanied by the final maturation cleavage of the capsid, i.e., autocatalytic conversion of VP0 to VP4 and VP2. However, in some picornaviruses, VP0 remains unprocessed and is present in the mature virion (Table 1) [70,71,72] while, in assembled FMDV empty capsids, VP0 processing can occur even in the absence of RNA packaging [73,74]. Progeny virions are normally released by cell lysis but may also be released from intact cells via a non-lytic process that involves release of infectious virus particles in membrane enclosed vesicles [75,76,77].

Picornavirus infection can inhibit cellular protein secretion, which may contribute to evasion of antiviral immunity [78]. Virus infections activate cellular antiviral defense mechanisms and picornaviruses have evolved a number of strategies to interfere with these responses [79,80,81]. In addition, viral proteins are known to enter the nucleus and inhibit host-cell transcription, which contributes to evasion of the innate cellular antiviral response [82]. Picornaviruses also disrupt nucleo-cytoplasmic trafficking [83,84,85]. This allows exploitation by these cytoplasmic viruses of proteins that normally reside within the nucleus, such as RNA-binding proteins and proteins that drive mobilization of neutral lipids that are required to generate RO [82,86].

## 4. Picornavirus 3A Proteins

**Figure 1:**
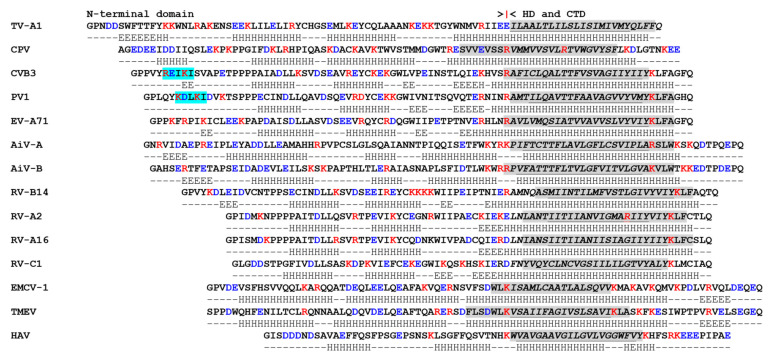
3A protein alignment for selected picornaviruses. Alignment of full-length 3A proteins for selected picornaviruses; Enteroviruses, poliovirus 1 (PV1) (Enterovirus C), coxsackie virus B3 (CVB3) (Enterovirus B), enterovirus A71 (EV-A71) (Enterovirus A), rhinovirus A2 (RV-A2) and rhinovirus A16 (RV-A16) (Rhinovirus A), rhinovirus B14 (RV-B14) (Rhinovirus B), rhinovirus C1 (RV-C1) (Rhinovirus C); Kobuviruses, AiV-A1 (Aichivirus A), AiV-B1 (Aichivirus B); Cardioviruses, encephalomyocarditis -1 (EMCV-1) (Cardiovirus A), Theiler’s murine encephalitis virus (TMEV) (Cardiovirus B); Hepatovirus, hepatitis A virus (HAV) (Hepatovirus A); Limnipivirus, carp picornavirus (CPV) (Limnipivirus B); Teschovirus, TV-A1 (Teschovirus A). 3A proteins have a similar organisation that consists of a N-terminal domain, a hydrophobic domain (HD) that includes a predicted membrane-binding region (MBR), followed by a C-terminal domain (CTD). Here, the HD (shown in italics) is loosely defined as a region devoid of charged aa (note for RV-A2 and CPV a single R residue may be present in the HD). The 3A sequences are shown using the single letter aa code and were aligned using the first charged aa (marked by **|**) before the putative HD (note, for TV-A1 there is no charged aa after the HD). Charged aa are shown in bold red (R and K) or bold blue text (D and E). Transmembrane binding regions were predicted using TM-PRED [90], TMHMM [91], PHOBIUS [92] and HMMTOP [93]. Grey boxes highlight the aa with the potential to form an MBR identified by at least one of the programs. For some viruses, some programs predicted that the MBR extended beyond the charged aa that flank the HD. Amino acids that were identified by all four programs are underlined and are likely to be involved in forming an MBR. For PV this includes the residues 65-AVTTFAAVAGVVYVMY-80 that were determined experimentally as the MBR by [94]. Note for EMCV-1 and CPV only 3 (TM-PRED, HMMTOP and PHOBIUS) and 1 (TM-PRED) program, respectively, predicted an MBR and therefore, for these viruses, no underlined sequences are shown. The residues that form the Golgi Brefeldin A Resistant Guanine Nucleotide Exchange Factor 1 (GBF1) binding site (residues 6-10 for CVB3 and PV) [95,96] are boxed in light blue. Note that these residues are mostly absent for RV-A2 and RV-A16, and the conserved K-9 (CVB3 and PV) is E-8 in RV-B14. Secondary structure predictions were made using Jpred4 and are indicated under each sequence (H = Helix, E = extended). Accession numbers of the sequences shown: PV1 V01149, CVB3 M88483, EV-A71 MG672481, RV-A2 X02316, RV-A16 L24917, RV-B14 K02121, RV-C1 EF077279, AiV-A AB040749, AiV-B AB084788, EMCV-1 AY296731, TMEV X56019, HAV M14707, CPV KF306267, and TV-A1 AJ011380.

**Figure 2:**
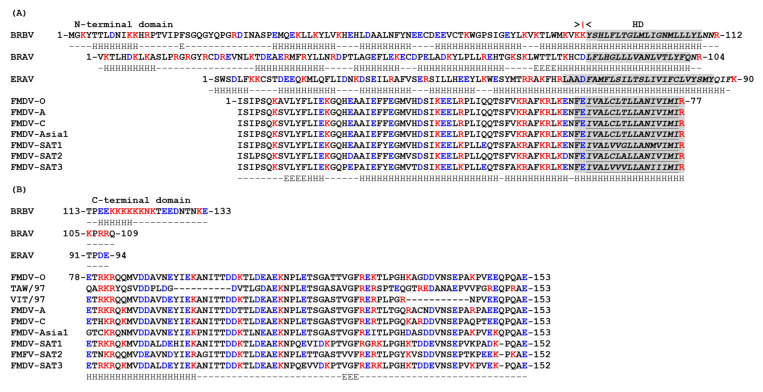
3A protein alignment for selected aphthoviruses. (**A**) Alignment of the 3A N-terminal domains and HDs of selected aphthoviruses (all seven serotypes of FMDV, namely FMDV-O, -A, -C, -Asia1, -SAT1, -SAT2 and -SAT3, plus equine rhinitis A virus (ERAV), bovine rhinitis virus A and B (BRAV-1 and BRBV-1). The 3A proteins have a similar organisation that consists of a N-terminal domain, a HD that includes a predicted MBR, followed by a CTD. The sequence annotation and alignment are as described for Figure 1. For BRAV-1 only 3 (TM-PRED, HMMTOP and PHOBIUS) programs predicted an MBR and therefore no underlined sequences are shown. Secondary structure predictions were made using Jpred4 and are indicated under each sequence (H = Helix, E = extended). Very similar secondary structures were predicted for each FMDV serotype and are represented by FMDV-SAT3. (**B**) Alignment of the 3A C-terminal domain of selected aphthoviruses (as described for panel A). In addition, the CTDs for FMDV, O/TAW/97 (TAW/97) and O/VIT/2/97 (VIT/97) [87,97] are included to show the position of the internal deletions; the deleted residues are indicated by – (residues 93–102 for O/TAW/97 and 133–142 for O/VIT/2/97). The CTDs of the FMDV-SAT serotypes have one residue less than the other FMDV serotypes. The missing residues are indicated by -. Accession numbers of the sequences shown: FMDV-O AJ539141, FMDV-A AY593752, FMDV-C AY593805, FMDV-Asia1 DQ533483, FMDV-SAT1 AY593840, FMDV-SAT2 AY593849, FMDV-SAT3 AY593850, O/TAW/97 AF308157, O/VIT/2/97 AJ295002, ERAV DQ272578, BRAV-1 KP236128, and BRBV-1 EU236594.

The 3A proteins of different picornavirus genera vary considerably in length and sequence (Figure 1 and Figure 2) and are therefore often omitted from phylogenetic analyses [1]. Despite being highly divergent, most 3A proteins have a shared overall architecture and often have relatively large N-terminal domains with relatively short C-terminal domains that are separated by a single hydrophobic region (or hydrophobic domain (HD)). The N-terminal domains vary in length (e.g., 89 residues, bovine rhinitis B virus 1 (BRBV-1) and 39 residues, hepatitis A virus (HAV)) and are usually longer than the C-terminal domains (Figure 1 and Figure 2). A notable exception is the 3A protein of FMDV where the C-terminal domain is longer (75–76 residues) than the N-terminal domain (ca. 60 residues), and considerably longer than in other picornaviruses [87]. Across the seven FMDV serotypes, the N-terminal region and the HD are highly conserved (Figure 2); however, FMDV 3A is the least conserved nsp at the amino acid (aa) level [87,88] due to high sequence diversity within the C-terminal domain. The function of the extended C-terminal domain of FMDV 3A is unknown but it may be involved in determining host range (see below). For most picornaviruses the HD can be readily assigned (Figure 1 and Figure 2) (this is discussed in more detail below). Although the 3A protein of aichi virus A (AiV-A), bovine kobuvirus (AiV-B) and human klassevirus (Salivirus A) are myristoylated at the N-terminal glycine (despite not having a canonical myristoylation motif) [89], 3A proteins are typically not post-translationally modified. 

The 3A protein is the N-terminal part of the P3 precursor (3ABCD). For PV, processing of P3 occurs by two distinct pathways (major and minor). The major pathway generates 3AB and 3CD^pro^ [98], while the minor pathway initially results in 3A and 3BCD [99,100]. The 3AB and 3CD^pro^ precursor proteins are relative stable in infected cells and processed slowly to their mature protein constituents, 3A and 3B, plus 3C^pro^ and 3D^pol^, respectively [101,102,103]. Thus, although 3A and 3AB are both present within infected cells, 3A is less abundant than 3AB especially during the early phase of infection. 

**Figure 3:**
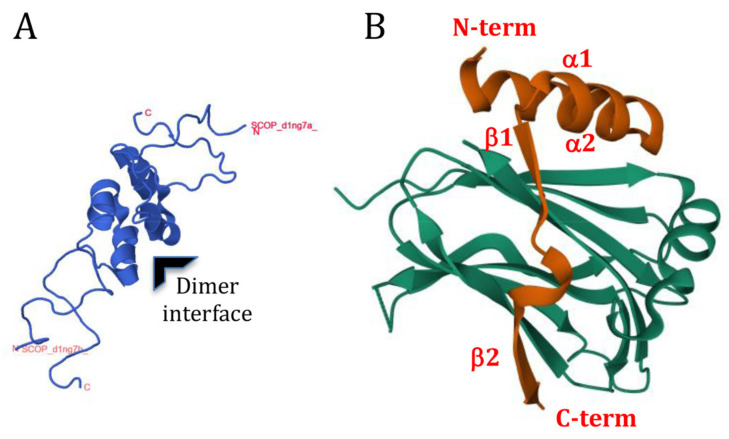
Structure of PV 3A. (**A**) The NMR structure [104] of a PV 3A (aa 1–59) dimer shown as a cartoon representation (PDB 1NG7). The central region of each 3A forms two amphipathic α helices Table 1. PV 3A (red) in a complex with the Golgi Dynamics (GOLD) domain of ACBD3 (green). Electron density could not be assigned for residues 1–14 of 3A. (**B**) In the complex, 3A formed four secondary elements (two α helices, α1, aa 19–29, and α2, aa 32–41, and two β strands, β1, aa 44–46, and β2, aa 53–58), which all make contacts with the GOLD domain (PDB 6HLV). 3A is wrapped around the GOLD domain so that the N- and C-termini of the N-terminal domain make contacts on opposite sides [107]. As in the nuclear magnetic resonance (NMR) structure, hydrophobic residues of α1 and α2 interact with the hydrophobic residues of the other 3A molecule that form the 3A dimer interface. When bound to ACBD3, dimerization of 3A drives formation of a 3A:ACBD3 heterotetramer. This allows for the membrane binding regions of the 3A molecules to “staple” ACBD3 to membranes at the RO (see [107] for details).

The solution structure of a part of PV 3A (aa 1–59), as determined by nuclear magenetic resonance (NMR) spectroscopy (Figure 3A), shows that the central region of this N-terminal domain forms two amphipathic α helices (aa 23-29, and 32–41) that are joined by a short connecting loop to form a “hairpin” structure [104]. The first 14 and approximately the last 11 residues (aa 48–59 in full-length 3A) of this molecule were unstructured, although it was noted that these regions could potentially form ordered structures when bound to other viral or cellular proteins [104]. In the solution structure, the 3A protein formed dimers with the interface formed by a hydrophobic surface consisting of residues Ile-22, Leu-25, Leu-26, Val-29, Val-34, Tyr-37, Cys-38 and Trp-43. Molecular modelling (based on the NMR structure of PV 3A) suggested that the N-terminal domains of the 3A proteins of FMDV and CVB3 also form dimers and that dimerization is required for vRNA replication [105,106]; thus, it is possible that the ability to form homodimers may be a common feature of picornavirus 3A proteins. 

## 5. Viral Binding Partner Proteins

Evidence for interactions involving 3A and 3AB with other viral nsps (including precursor proteins) primarily comes from studies with PV and AiV using yeast-2-hybrid (Y-2-H) and mammalian-two-hybrid (M-2-H) systems, and from biochemical studies and the use of recombinant viruses [108,109,110,111,112]. Collectively, these studies have shown that although 3A and 3AB likely interact with several different nsps they have different binding profiles, which is consistent with the notion that 3A and 3AB have different roles during picornavirus replication. The protein interaction study results are summarized in Table 2.

### 5.1. Dimerization of PV 3A and 3AB

In both the Y-2-H and M-2-H systems, PV 3A was shown to form homodimers andalso to bind to 3AB, whereas 3AB homodimers were only observed in the Y-2-H system and not in the M-2-H system [110,111]. As described above, the NMR structure and mutagenesis studies (and more recent structural analysis; see below) support the case for 3A homodimerization.

### 5.2. Interactions between PV 3A/3AB and the P2 Proteins (2B, 2C and 2BC)

The 3A and 3AB proteins were shown to interact with 2B in both the Y-2-H and M-2-H systems [110,111,112]. Interactions between 3A/3AB and 2B may be functionally important, as defective replication due to either aa substitutions within the HD, or the insertion of epitope tags into the N-terminal region of 3A, could be rescued by compensatory changes within 2B [113,114].

The 2C protein was identified as a binding partner for 3A in both the Y-2-H and M-2-H systems (see Table 2) but only interacted with 3AB in the Y-2-H system [110,111]. However, an interaction between 2C and 3AB has been demonstrated in GST pull-down experiments using purified conjugated proteins [111]. This study also suggested that monomeric 3AB, and higher order 3AB homo-structures, may have distinct functions during PV replication, as multimerization of 3AB appeared to reduce binding to 2C [111]. Evidence for a functional interaction between 2C and 3A during PV replication comes from studies using recombinant PV in which the N-terminal residues of 2C were replaced by the corresponding residues of RV-B14 2C [110]. These viruses replicated poorly; however, near wild-type (wt) levels of replication were restored as a result of substitutions in 3A [110]. 3A and 3AB are also reported to interact with the 2BC precursor in the M-2-H system [110]. However, despite positive interactions involving 3A and 3AB with 2B and 2C (Table 2), binding of 3A and 3AB to 2BC was not detected in the Y-2-H system [110,112].

### 5.3. Interactions between PV 3A/3AB and 3D^pol^, 3CD^pro^ and 2A^pro^

In the Y-2-H system, 3AB, but not 3A, interacted with 3D^pol^ [108,111]. Substitutions in the 3A region of 3AB that impaired 3AB homodimerization did not adversely affect the interaction with 3D^pol^, which suggests that homodimerization may not be important for 3AB binding to 3D^pol^. The ability of 3AB (but not 3A) to bind to 3D^pol^ is consistent with other studies (see below) that show that 3AB (but not 3A) can stimulate RNA synthesis by 3D^pol^.

Structural studies have identified three distinct 3B-binding sites on 3D^pol^ [115,116,117]. Interestingly, two such sites do not involve residues at the active site of 3D^pol^ and it has been suggested that when 3B is bound at these sites it could be uridylylated, *in trans,* by another 3D^pol^ molecule [115,117]. Alternatively, 3B could have a stabilizing role within the uridylylation complex [115,117,118] and it is possible that the 3B binding sites that lie outside of the active site could be preferentially occupied by 3B when part of 3AB.

3AB also interacted with 3CD^pro^ in the Y-2-H system [108,111] and, although the binding appeared weak, this interaction was confirmed in a far-Western blot assay [111]. In addition, 3AB has been shown to bind 3D^pol^ and 3CD^pro^ in immunoprecipitation experiments [119]. Furthermore, the ability of 3AB to bind and form a complex with 3CD^pro^ and the CL at the 5′ end of the PV genome is central to formation of the viral RC and this is discussed below. Additionally, in the Y-2-H system PV 3A, but not 3AB, was shown to interact with 2A^pro^ [112].

### 5.4. Binding Partners of AiV 3A

In an M-2-H assay, AiV 3A interacted with itself and with 3AB (Table 2). In addition, it bound to 2A, 2B, 2C and 2BC but not to 3C^pro^, 3D^pol^ or the 3CD^pro^ precursor [109]. 3AB also interacted with 2C and 2BC, however, in contrast to 3A, 3AB did not appear to form homodimers or interact with 2B. Although 3A did not interact with 3C^pro^, 3D^pol^ or 3CD^pro^, the AiV 3AB did interact with 3C^pro^ and 3CD^pro^ (but not with 3D^pol^), which suggests that, as with PV, an interaction between 3AB and 3CD^pro^ may be central to forming the AiV RC [120]. Other notable differences in the binding partners of PV and AiV 3AB are: (i) in the M-2-H assay PV but not AiV 3AB interacted with 2B, (ii) AiV (in the M-2-H assay) but not PV (in the Y-2-H assay) 3AB interacted with 3C^pro^, and (iii) PV (in the Y-2-H) but not AiV (in the M-2-H assay) 3AB interacted with 3D^pol^ [109,110].

## 6. Inhibition of Protein Secretion

Some picornaviruses, (e.g., PV, CVB3 and FMDV) inhibit protein trafficking through the cell [78,121,122]. This suppresses cell-surface expression and secretion of proteins involved in the host antiviral response [78,79,122,123,124,125]. This process appears to be an advantage in vivo as a recombinant CVB3 incapable of inhibiting protein trafficking was less virulent in mice despite showing near normal replication in cultured cells [95]. For PV and CVB3, inhibition of protein trafficking can be recapitulated by expression of 3A alone [78,122]. However, the ability of 3A to inhibit protein trafficking is not universally shared among picornaviruses, as it is 2BC of FMDV, and not 3A, which inhibits protein trafficking [121,126]. Moreover, the 3A proteins of EV-A71, RV (RV-A2 and RV-B14), Theiler’s murine encephalomyelitis virus (TMEV), encephalomyocarditis virus (EMCV) and HAV do not have an inhibitory effect [127,128]. In contrast to RV-A2 and RV-B14, the 3A protein of RV-A16 was reported to inhibit protein trafficking; however, protein secretion is not inhibited in RV-A16-infected cells and the reason for this apparent disparity is unknown [129].

A number of studies have investigated how 3A of enteroviruses (CVB3 and PV) inhibit protein trafficking [78,128,130]. Collectively, these studies show that 3A blocks anterograde transport between the endoplasmic-reticulum (ER)-Golgi intermediate compartment (ERGIC) and the Golgi by preventing formation of coatomer protein complex I (COPI)-coated transport vesicles. Before describing how 3A is thought to inhibit the secretory pathway, it is worth briefly describing how COPI-coated transport vesicles are formed in the Golgi. The assembly of COPI-coated vesicles is initiated by activation of ARF1 (ADP-ribosylation factor 1) [131]. In the Golgi, activation of ARF1 is triggered by GBF1 (Golgi Brefeldin A Resistant Guanine Nucleotide Exchange Factor 1). As its full name indicates, GBF1 is a guanine nucleotide exchange factor (GEF) that catalyzes GDP/GTP exchange and converts ARF1-GDP (the inactive form) to ARF1-GTP (the active form) [132]. The COPI coat (or coatomer) is then recruited by a direct interaction with ARF1-GTP, and then COPI-coated vesicles subsequently bud from the Golgi. ARF GTPase-activating proteins (GAPs) interact with ARF1-GTP and induce GTP hydrolysis to generate ARF1-GDP in a process that may be enhanced by the COPI coatomer [133]. Normally, GBF1 cycles on and off membranes and each membrane-binding event triggers GDP/GTP exchange and, hence, the activation of ARF1 [133]. Activated ARF1 also shows increased membrane binding, thus, increased membrane association of GBF1 and ARF1 are normally considered to indicate conversion to their active states.

Initial evidence indicating how CVB3 inhibits protein trafficking came from the observations that the N-terminal (aa 1–60) domain of CVB3 3A binds to GBF1 (in a Y-2-H assay and in pull down experiments) and could inhibit its GEF activity [95,96]. Furthermore, expression of 3A reduced cellular levels of activated ARF1 (ARF1-GTP) and this could be overcome by co-expression of GBF1 (along with 3A) [95]. The role of 3A in these studies was confirmed by using a serine-insertion modification in 3A immediately after residue 15 (in CVB3 called 3A-ins 16S). This insertion prevents 3A binding to GBF1 and impairs the ability of 3A to: (i) inhibit the GEF activity of GBF1, (ii) reduce cellular levels of ARF1-GTP and (iii) inhibit protein trafficking [95,96]. Similar observations to those described above for CVB3 were made for PV 3A, which suggested that their 3A proteins inhibit protein trafficking by the same mechanism [128].

The binding site for GBF1 has been mapped to the N-terminal region of enterovirus 3A and deletion of the first 10 N-terminal residues renders 3A incapable of inhibiting protein trafficking [95,124,128]. Dimerization is required for 3A to inhibit protein trafficking [106] and substitutions in 3A that prevent dimerization [106,125] also prevent binding to GBF1 [96]. However, other substitutions in 3A that prevent binding to GBF1 and the ability of 3A to inhibit protein trafficking do not affect dimerization [96,106]. Based on these observations it was suggested that 3A dimerization may be required to present the GBF1-binding site to 3A [96].

In addition to showing that the 3A proteins of RV-A2 and RV-B14 fail to inhibit protein trafficking, Wessels et al. [128] showed that they have a reduced ability to interact with GBF1 due to aa differences within the identified GBF1-binding site (see Figure 1). 3A of RV-A2 did not interact with GBF1 in a Y-2-H assay whereas 3A of RV-B14 made only weak interactions [128]. This suggested that the most likely explanation for the failure of the 3A proteins of RV-A2 and RV-B14 to inhibit protein trafficking was an inability or reduced ability to bind GBF1. As noted above, the 3A protein of RV-A16 was reported to inhibit protein trafficking despite the secretory pathway functioning normally in RV-A16-infected cells [129]. Interestingly, as for RV-A2, the 3A protein of RV-A16 also lacks the residues in the N-terminal region that form the GBF1 binding site (Figure 1), and therefore would not be expected to bind to GBF1.

Interestingly, the study by Wessels et al. [95] showed, in FRAP (fluorescence recovery after photo-bleaching) experiments, that membrane association of GBF1 was increased in 3A-transfected cells. This suggested that GBF1 could be activated by 3A. However, Brefeldin A (BFA) inhibits ARF1 activation by forming an inactive complex between GBF1 and ARF1-GDP and thereby stabilizes GBF1 on membranes [134]. Thus, the increased membrane association of GBF1 induced by 3A could indicate that (similar to BFA) 3A inhibits GBF1 when it is membrane associated [95].

Although the 3A proteins of PV and CVB3 inhibit protein secretion, it is not clear if this is also the case for 3AB. An early study reported that PV 3AB does not have an inhibitory effect on protein trafficking [78]. In contrast, a more recent report indicated that PV 3AB can inhibit protein trafficking, but to a lesser extent than 3A [135]. These observations suggest that 3AB may not be able to bind to GBF1 or may bind with reduced efficiency compared to 3A. However, problems with the expression of 3AB have been reported [124] and whether PV 3AB can bind to GBF1 and inhibit protein trafficking is currently not clear.

As discussed further below, picornavirus 3A proteins (and certain 3A-containing precursor proteins) have an important role(s) in vRNA replication. However, the roles of 3A in vRNA replication and in inhibiting protein trafficking appear distinct, as aa substitutions have been identified in enterovirus 3A proteins that attenuate infection but do not reduce the ability of 3A to inhibit protein trafficking [95,106,124,127]. Conversely, other changes have been identified that abrogate the ability of 3A to inhibit protein trafficking but do not prevent virus replication [95,106,122,123,124]. One such mutant is the 3A-ins 16S serine-insertion described above for CVB3 3A. In PV, this insertion mutant is called 3A-2 and, similar to CVB3, the modification results in viable viruses that are unable to inhibit protein trafficking [95,124,136].

The above studies have established that the 3A proteins of PV and CVB3 bind to GBF1 and that CVB3 3A inhibits GBF1 activation. However, enterovirus replication requires the type III phosphatidylinositol 4-kinase (PI4K), PI4KIIIβ (from here onwards termed PI4KB) (see below), and the interaction between 3A and GBF1 is thought to provide a mechanism (via GBF1 and ARF1 activation) to recruit PI4KB to the RC. These seemingly contradictory outcomes of 3A binding to GBF1 have fueled an on-going conundrum regarding the roles of 3A and GBF1 in enterovirus replication, which is discussed in more detail below.

## 7. Membrane Interactions

3A and 3AB are membrane-binding proteins and PV 3A/3AB interacts with membranes when expressed in the absence of other viral proteins [94,102,137,138,139,140]. Figure 1 and Figure 2 show an alignment of 3A sequences of selected picornaviruses. Although 3A proteins are highly charged, a region devoid of charged residues can be identified towards the C-terminus of the protein, which is consistent with a HD (see above). However, for teschovirus A1 (TV-A1) the HD appears to extend to the C-terminus of the protein (Figure 1). The HD of PV 3A is comprised of 22 residues (59–80) and includes the region that anchors 3A/3AB to membranes (here called the membrane-binding region (MBR)). For PV 3A, the MBR has been mapped experimentally to residues 65–80 [94]. This is consistent with earlier studies that identified residues within the C-terminal half of the HD as being critical for PV 3A membrane association [140]. In line with these observations, residues 62–79 were predicted to be part of an MBR by four structure prediction programmes, TM-PRED [90], TMHMM [91], PHOBIUS [92] and HMMTOP [93] (Figure 1). These programs also predict the presence of an MBR for most of the 3A proteins shown in Figure 1. Interestingly, for Carp picornavirus 1 (CPV) only one of them, TM-PRED, predicted an MBR (Figure 1), which suggests that this 3A protein may make novel interactions with membranes.

The 3AB precursor is an efficient substrate for processing when membrane-associated [94]. Models of how 3AB interacts with membranes have considered the need to maintain the P3 proteins on the cytoplasmic side of the membrane, to facilitate viral polypeptide processing and formation of the viral RC [94,141]. Consistent with this, PV 3AB was shown to be orientated parallel to the membrane bilayer such that it only interacts with the outer leaflet (i.e., adopts a monotopic orientation) [94]. Interestingly, the topology of membrane association was different for the mature 3A protein, which adopted two alternative orientations; a monotopic orientation (as described for 3AB) and a bitopic orientation [94]. The 3B peptide is hydrophilic and effectively extends the length and increases the hydrophilic nature of the C-terminal domain of 3A, and it is likely that this blocks the ability of 3AB to fully span the membrane [94]. Furthermore, the observations described above suggest the possibility that a switch between monotopic and bitopic membrane associations may occur on processing of PV 3AB to 3A and 3B [94].

The 3A protein of FMDV is considerably longer (153 residues) than other picornaviruses and has an extended C-terminal domain [87] (Figure 2). However, like other picornaviruses, FMDV 3A has three clearly defined regions; N- and C-terminal domains separated by a HD (residues 61–76). The sequence of the N-terminal and HDs are very highly conserved across FMDV serotypes while, in contrast, the C-terminal domains show rather limited sequence identity [87]. As for PV, the HD of FMDV 3A includes an MBR [142]. The substitution of residues 59 to 76 by a shorter sequence of 8 consecutive Ala residues abrogated FMDV replication, which suggests that the membrane association of FMDV 3A is important for vRNA replication [143] (however, this is a fairly major modification which could influence the protein properties in a variety of ways). Unlike the PV 3A that can fully span cellular membranes, FMDV 3A has been shown to adopt a monotopic orientation on membrane binding [142]. Presumably this is because FMDV 3A has a longer C-terminal region that prevents a bitopic membrane association. Thus, due to its longer C-terminal domain, removing 3B from FMDV 3AB may not have the same effect of switching from monotopic to bitopic membrane binding.

## 8. Host-Range Determinant

Some picornaviruses have a restricted host range (e.g., PV) [144], while others such as FMDV have a broad host range that includes economically important livestock (e.g., cattle, buffalo, sheep, goats and pigs) and numerous cloven-hoofed species of wild animal [145,146]. Amino acid changes in picornavirus 3A proteins can alter the host range of the virus. For example, aa substitutions in 3A allow replication of RV-A1 in mouse cells [147,148] and increase replication of RV-C clinical isolates in cell culture [149]. Changes in 3A have also been implicated in improved growth in cell culture of hepatoviruses [150,151,152,153] and have been reported to influence tissue tropism of CVB3 in mice [154].

Substitutions in FMDV 3A are also associated with altered host range. A Gln-44-Arg substitution in the N-terminal domain of the 3A protein of FMDV C-S8c1 allows infection of guinea pigs [155]. The mechanisms that underlie how this change results in replication in guinea pigs’ cells are unknown. The Gln-44 residue is conserved across all FMDV serotypes and lies adjacent to the predicted interface between 3A molecules of a 3A-homodimer where it could make interactions with other viral proteins or with cellular proteins that are involved in FMDV replication. Deletions within the C-terminal domain of FMDV 3A are also linked to determination of host range. Deletion of residues 84–102 in O1Campos O/E, or residues 88–106 in C3 Resende/ Brasil55 were first linked to host specificity as they were lost in viruses that were attenuated for cattle (but not pigs) after passage through chicken embryos during vaccine development [156,157,158,159]. A similar deletion in 3A (aa 93–102) was found in a natural field isolate (FMDV O/YUN/TAW/97) that caused an outbreak of FMD in Taiwan in 1997 [160,161]. This outbreak only affected pigs, which suggested that the O/YUN/TAW/97 virus could be attenuated for cattle. In line with this, subsequent studies appeared to link this deletion with attenuation in cattle since recombinant chimeric viruses (based on O/YUN/TAW/97 and a bovine-virulent FMDV) showed that viruses with the 93–102 deletion in 3A had restricted replication in primary bovine cells and were attenuated for cattle [160]. Further studies suggested that the 93–102 deletion in 3A (and a similar deletion of residues 87–106) reduced replication in bovine cells without affecting replication in porcine cells, or virulence for pigs [87,97,162,163]. However, analyses of type O FMDVs circulating in South-east Asia (between 1970–1999) identified viruses with the 93–102 deletion in 3A that were initially isolated from cattle; and furthermore, some of these viruses (e.g., O/HKN/21/70) replicated similarly to wt viruses in bovine and porcine primary keratinocytes [87]. This suggested that the 93–102 deletion in 3A may not be solely responsible for the poor replication of O/YUN/TAW/97 in bovine cells. As pointed out by the authors [87], the 3A protein of O/YUN/TAW/97 also had accumulated a large number of other aa substitutions in the C-terminal domain that could also contribute to the inability of O/YUN/TAW/97 to replicate in bovine cells. The study by Pacheco et al. [97] appeared to show that the 87–106 deletion in 3A did not abrogate FMDV replication in an established bovine cell line (LFBK) that had been transfected to express the FMDV receptor, integrin αvβ6. However, subsequent analysis has shown that these LFBK cells were actually of porcine origin and the ability of FMDV with the 87–106 deletion in 3A to replicate in bovine cell lines remains to be determined [164].

The FMDVs circulating in South-east Asia [87] also had a second deletion in the C-terminal domain of FMDV 3A that spanned residues 133-143. Similar to the viruses with the 93–102 deletion, viruses with the 133–143 deletion in 3A replicated well in porcine cells [163]. However, the effect of this deletion on FMDV replication in bovine cells is less clear. Two studies have reported that FMDV with the 133–143 deletion could replicate in bovine primary keratinocytes and primary bovine fetal kidney cells [87,165] whereas another study found that replication in secondary bovine kidney cell cultures is severely attenuated [163]. Based on these natural deletions, a recombinant virus with a more extensive deletion in 3A (lacking 52 residues, aa 93–143; so-called 3A super-deleted) has been generated and shown to display severely reduced replication in fetal bovine kidney cells [163]. Interestingly, viruses with this deletion replicate with wt kinetics in fetal porcine kidney cells [163], and caused severe FMD symptoms in pigs, which suggests that almost the entire C-terminal domain of 3A is not required for FMDV replication in porcine cells.

FMDV with the 87–106 deletion in 3A has been studied in more detail. In vivo studies have confirmed that viruses with this deletion are virulent for pigs [97] but attenuated for cattle [97,166]. Following aerosol inoculation, cattle infected with FMDV carrying this deletion in 3A displayed few or no clinical signs of FMD and did not develop viremia or generate neutralizing antibodies [166]. Infectious virus and vRNA were detected in the pharyngeal areas at day 21 post-inoculation [166] suggesting that vRNA replication had occurred but was restricted to these areas. In addition, similar mRNA levels encoding several anti-viral cytokines in nasopharyngeal tissues were seen in animals infected with either the wt virus or the virus with the deletion in 3A. These findings lead the authors to suggest that cattle attenuation of FMDV resulting from the 87–106 deletion in 3A more likely results from reduced intracellular virus replication, rather than differences in the host anti-viral response. However, it is possible that cattle attenuation due to the 87–106 deletion in 3A results from the need for 3A to combat the antiviral effects of as yet unidentified host restriction factor(s) that function in bovine but not porcine cells.

## 9. Nucleic Acid Chaperone Activity

PV 3AB displays characteristics of a nucleic acid chaperone and promotes (i) sequence-independent nucleic acid (DNA and RNA) hybridization and (ii) unwinding of nucleic acid secondary structure [167]. The ability of 3AB to enhance hybridization appears to require 3A to coat the target nucleic acid. The 3B peptide alone has no activity; however, the chaperone activity of 3AB appears to be dependent on the 3B region, as substitutions within 3B abrogated this function [168]. Consistent with these observations, a peptide consisting of 3B and the 7 aa from the C-terminal domain of 3A (peptide 3B+7) could mimic the chaperone activity of full-length 3AB indicating that the chaperone activity of 3AB resides collectively within the C-terminal domain of 3A and 3B [168]. More recently, the same region of EV-A71 3AB has been reported to display RNA chaperone (destabilizing RNA helices and stimulating RNA strand annealing) activity [169]. It is not clear how the chaperone role of 3AB contributes to enterovirus replication. However, as discussed [167], nucleic acid chaperone activity could enhance recombination or facilitate vRNA replication by promoting unwinding of vRNA templates and/or destabilizing secondary structures and could facilitate packaging.

## 10. Interactions with Cellular Proteins

Picornavirus 3A proteins have been reported to interact (directly or indirectly) with a number of cellular proteins. However, for most of these interactions, the precise role of the cellular protein in facilitating virus replication is currently unclear. The functional consequences for protein secretion resulting from the binding of the 3A proteins from PV and CVB3 to GBF1 have been described above. However, GBF1 is also required for enterovirus replication and the role of 3A in recruiting GBF1 to the RC is described in detail below. Similarly, 3A interactions with ACBD3 (Acyl-CoA binding domain containing protein 3), type III phosphatidylinositol 4-kinase (PI4K) and lipid droplet enzymes are associated with vRNA replication and these are also described below. In the next part of the review, we describe interactions between 3A and cellular proteins that do not appear to directly influence vRNA replication.

### 10.1. LIS1

Poliovirus 3A has been shown to bind LIS1 (Lissencephaly 1 Protein) in a Y-2-H assay and in pull-down experiments using tagged proteins [170]. LIS1 promotes formation of the dynein-dynactin complex and thereby activates dynein [171,172]. Dynein 1 moves diverse cargos (including organelles, vesicles and RNA) along microtubules and contributes to maintaining the distribution, organization, structural integrity and functions of many cellular organelles [173,174]. The role of LIS1 in PV infection has not been investigated but it has been suggested that binding to LIS1 could contribute, in part, to inhibition of protein trafficking by 3A, or could be involved in non-lytic virus release [76]. However, an independent study failed to detect LIS1 as a 3A-binding partner in 3A pull-down experiments [113]. This study used recombinant PV with insertion tags near the N-terminus of 3A; thus, it is possible that such tags impair 3A binding to LIS1. However, given that viruses with 3A insertion tags were viable, it was suggested that LIS1 may not be required for vRNA replication per se but could serve to promote infection in vivo [113].

### 10.2. Dynactin-3

Dynactin-3 is a subunit of the dynactin complex (that serves as a cofactor for dynein) and is essential for most cellular functions carried out by dynein. The 3A protein of FMDV O1 Campos has been reported to bind to dynactin-3 in both Y-2-H and M-2-H assays [175]. However, attempts to confirm the interaction between 3A and dynactin-3 in FMDV-infected cells using immunofluorescence confocal microscopy were inconclusive. In Y-2-H experiments, residues within the C-terminal domain of 3A were shown to mediate binding to dynactin-3. Specifically, changing residues 89-Ala-Val-Asn-Glu-92 (89-AVNE-92) to 89-Pro-Leu-Asp-Glu-92 (89-PLDG-92) abrogated the interaction between 3A and dynactin-3. The PLDG motif was chosen as a substitute for 89-AVNE-92 because it is present at the corresponding site in 3A of FMDV O/TAW/97, which was shown not to interact with dynactin-3 in the Y-2-H system. A recombinant FMDV (based on O1 Campos) including the 3A 89-AVNE-92 to 89-PLDG-92 substitutions replicated poorly in primary bovine cell cultures, and only caused mild symptoms of FMD in cattle [175]. Sequencing of viruses isolated from secondary lesions of three cattle infected with the 3A PLDG virus revealed changes at the Pro residue to either Ala (one animal) or Leu (the other two animals). Consistent with these observations, an Ala-89-Leu substitution introduced into 3A of O1 Campos did not alter 3A binding to dynactin-3 in a Y-2-H assay, whereas the introduction of Pro at this site (Ala-89-Pro) prevented the interaction. The precise role of dynactin-3 in FMDV infection of cattle remains to be determined, but the failure of the 3A PLDG virus to replicate in primary bovine cells suggest that dynactin-3 may be required for FMDV replication. However, the recombinant FMDV including the 3A 89-AVNE-92 to 89-PLDG-92 substitution displayed near wt replication kinetics in a continuous porcine kidney cell line expressing αvβ6, which suggests that the interaction between 3A and dynactin-3 is not required for FMDV replication in porcine cells. Interestingly, this porcine kidney cell line supports replication of a leaderless FMDV, which suggests that it could have a defective interferon response [176]. Thus, it will be interesting to determine if the 3A PLDG virus can replicate in primary porcine cells and/or retains virulence for pigs. Interestingly, the virus with substitutions within the dynactin binding site has similar growth characteristics to viruses with internal deletions in the C-terminal domain of 3A (i.e., poor replication in primary bovine cells and cattle while retaining wt replication in porcine cells). The internal deletions removed the residues that form the dynactin-binding site, which suggests that the interaction between 3A and dynactin could contribute to the host range specificity of FMDV [175].

### 10.3. Vimentin

Vimentin is a major intermediate filament and provides a cytoplasmic scaffold for the organization and functions of cellular organelles, and other cellular processes such as cell spreading, migration and signal transduction [177]. In addition, vimentin has been reported to actively support viral infections [178]. FMDV 3A has been identified as a binding partner for vimentin from mass spectrometry analysis of proteins that co-purified with 3A (using an anti-3A antibody) from FMDV-infected primary fetal bovine kidney (FBK) cells [179]. The interaction was also demonstrated by the simultaneous pull-down (using anti-c-Myc agarose beads) of FLAG-tagged vimentin and Myc-tagged FMDV 3A from HEK293T cells co-expressing these proteins. However, labelling for 3A and vimentin showed limited overlap in FMDV-infected pig kidney cells. The interaction with vimentin was reported to involve residues 15–21 of 3A [179]. However, it is not clear what effect the changes introduced into 3A (i.e., residues 15–21 were replaced by 7 consecutive Ala residues) had on the overall structure of 3A. Recombinant FMDV with these changes in 3A could not be rescued suggesting a possible link between 3A binding to vimentin and FMDV replication; however, these changes would likely interfere with the other functions of 3A/3AB during virus replication. FMDV replication was inhibited by overexpression of FLAG-tagged vimentin or by disrupting vimentin with acrylamide, whereas siRNA knockdown of vimentin appeared to enhance FMDV replication. However, these studies need to be reconciled with the observations that vimentin is not normally expressed in epithelial cells, which are the cell type preferentially infected by FMDV in vivo [180,181].

## 11. Evasion of the Cellular Antiviral Response

The innate cellular antiviral response prevents replication of viruses to restrict their spread within the host. Detection of virus infection by the cellular antiviral response is dependent on pathogen-associated molecular patterns (PAMPs) within vRNA. In the cytoplasm, PAMPs are detected by pattern recognition receptors (PRRs), which “sense” vRNA. PRRs include the double-stranded RNA-dependent protein kinase (PKR) and the RIG-I-like receptors (RLRs), RIG-I (Retinoic acid-inducible gene I), MDA5 (Melanoma Differentiation-Associated protein 5) and LGP2 (laboratory of genetics and physiology 2). On binding to PAMPs, RLRs trigger signaling cascades through MAVS (Mitochondrial antiviral signaling protein; also known as VISA; Virus-Induced-Signaling Adapter) that culminate in activation of transcription factors (such as IFN regulatory factor 3 (IRF3), IRF7 and NF-kappa B (NF-қB). These factors then translocate to the nucleus and drive the expression of various antiviral molecules, including the interferons (INFs), which restrict virus replication and orchestrate the adaptive immune response [182,183].

To combat the cellular antiviral response, picornaviruses have evolved a number of strategies. These include: inhibition of host-cell protein synthesis and transcription, preventing nuclear translocation of transcription factors, or inducing degradation (either indirectly by proteasome- and caspase-dependent processes, or directly via virus-encoded proteases) of host proteins that sense infection [82,184,185,186,187]. HAV 3A has been implicated as having an indirect role in evading antiviral responses. HAV targets MAVS in infected cells. MAVS is localized to mitochondria and essential for the cellular antiviral immune response to RNA virus infection [188]. During infection by HAV, MAVS is degraded by the protease activity of 3C^pro^ when part of a larger 3ABC precursor, which associates with mitochondrial membranes via the membrane anchor region of 3A. In addition, a number of recent studies have reported that the 3A proteins of some picornaviruses contribute to evasion of the cell antiviral response by interacting with, or regulating expression of, some of the key proteins involved (see below).

### 11.1. ATP1B3

ATP1B3 (Sodium/potassium-dependent ATPase subunit beta-3) is a regulatory subunit of Na+/K+–ATPase [189]. Recently, ATP1B3 was proposed to positively regulate IFN expression by activating NF-қB [190,191]. In line with such a role, replication of EV-A71 was reduced by overexpression of ATP1B3 and enhanced by ATP1B3 knockdown [190]. This study also reported that EV-A71 3A could bind to ATP1B3 in a Y-2-H assay and in co-immunoprecipitation experiments using epitope tagged proteins expressed in transfected cells. Furthermore, the interaction between endogenous ATP1B3 and 3A was detected in EV-A71-infected human rhabdomyosarcoma cells [190], which suggests that 3A could interfere with the functions of ATP1B3 during infection.

### 11.2. RIG-I, MDA5 and MAVS

FMDV 3A has been reported to bind to RIG-I, MDA5 and MAVS in immunoprecipitation experiments [192]. This study also reported that mRNA and protein levels of RIG-I, MDA5 and MAVS were reduced in cells transfected to express 3A. In addition, 3A was reported to inhibit the IFN-β signaling pathway, and phosphorylation and dimerization of IRF3 induced by Sendai virus (SeV). However, this study used human cells and relied solely upon overexpression of 3A in isolation from other viral proteins, which may not completely reflect the function of 3A in evasion of the cell antiviral response. It would be of interest to determine if 3A interacts with RIG-1, MDA5 and MAVS in FMDV-infected cells derived from a natural host of FMDV. Moreover, the above observations need to be reconciled with similar studies that concluded that expression of FMDV 3A (or 3AB) does not influence expression of ISGs in HeLa cells following treatment with IFNβ [193], and more recent studies which showed that L^pro^ targets MDA5 for degradation in FMDV-infected porcine cells [186].

### 11.3. DDX56

The DEAD-box family protein, DDX56 has been proposed to down regulate the IFN signaling pathway by preventing the interaction between IRF3 and IPO5 (a nuclear transport receptor) and thereby reducing IRF3 nuclear import [194]. Recently, FMDV 3A has been reported to cooperate with DDX56 to inhibit the IFN signaling pathway by reducing IRF3 phosphorylation [195], which is also required for nuclear translocation and the antiviral functions of IRF3.

### 11.4. G3BP1

G3BP1 (Ras-GAP SH3-binding protein 1) is an antiviral protein and has been shown to be important for stress granule (SG) formation [196,197]. Assembly of SGs is an important host response to viral infection as they serve as signaling hubs that contribute to the cellular antiviral response [198]. Thus, many viruses including picornaviruses block their assembly during infection (reviewed in [199]). In addition to nucleating SG formation, G3BP1 has been shown to regulate the cell antiviral response via activation of PKR, JNK (Jun N-terminal kinase) signaling and transcription mediated by NFκB [197]. Furthermore, G3BP1 has been shown to interact with RIG-I and dsRNA to induce production of IFN [200]. Recently, FMDV 3A has been reported to interact with porcine G3BP1 [201]. This study also reported that expression of 3A alone induced degradation of G3BP1 by a process that involved LRRC25 (Leucine Rich Repeat Containing 25) and possibly autophagic degradation [201]. However, these observations need to be considered against separate reports that provided evidence that the FMDV 3C^pro^ [202,203] or alternatively the FMDV L^pro^ can cause degradation of G3BP1 in FMDV-infected porcine cells [204].

### 11.5. RNA Interference

RNA interference (RNAi) is a gene-silencing mechanism that is conserved through evolution in many diverse organisms and contributes to host defence against pathogens. Consequently, many viruses have evolved viral suppressors of RNAi (VSR) that inhibit the host RNAi silencing. However, the role of RNAi as a component of the cellular antiviral defense response in mammals has been the topic of debate [205]. Recently, the abilities of enterovirus 3A proteins to function as VSRs have been investigated and provided contradictory evidence and conclusions. A recent study has concluded that EV-A71 3A (and 3AB) functions as a potent VSR [206]. This study showed that FLAG-tagged 3A could suppress RNAi-mediated silencing of EGFP expression in HEK293T cells [206]. In addition, EV-A71 3A was shown to bind and protect long dsRNA from processing by Dicer (an RNase III enzyme that processes long precursor dsRNAs to short dsRNAs). A single aa substitution (Asp-23-Ala) (D23A) abrogated the ability of 3A to bind and protect dsRNA from Dicer and also reduced EV-A71 replication. Together these observations suggested that an RNAi response could be triggered on infection by EV-A71 with the 3A D23A mutation and that this could restrict its replication. Consistent with this conclusion, replication of EV-A71 carrying the 3A D23A change could be partially rescued on infection of dicer-deficient cells, or by ectopic expression of wt 3A but not by 3A with the D23A change. Furthermore, EV-A71-derived small RNAs with characteristics of canonical siRNA (~22 nt long and derived from both strands of the EV-A71 vRNA and enriched for the 5’ and 3’ ends of the vRNA) could be detected in cells infected by EV-A71 carrying the 3A D23A substitution, but not after infection with the wt virus. This suggested that EV-A71 3A could potently inhibit RNAi. Following this study, the 3A protein of CVB3 was also reported to inhibit RNAi [207]. This study also identified a number of residues (24-Asp-Leu-Leu-26, 37-Tyr-Cys-38 and Arg-60) that are important for CVB3 3A binding to dsRNA and to suppress siRNA production. Interestingly, these residues are present (although in slightly different positions due to the different lengths of their 3A proteins) in 3A of EV-A71 and other enteroviruses (including PV, see Figure 1), which suggested that the ability of 3A to suppress RNAi could be common among enteroviruses.

In contrast to the above findings, myc-tagged 3A of EV-A71, CVB3 and PV were shown not to suppress RNAi-mediated silencing of firefly luciferase in transfected HeLa cells [208]. In addition, this study showed that infection of HeLa and HEK293T cells by a replication-defective CVB3, with a D24A substitution (equivalent to the D23A change in EV-A71), did not generate canonical virus-derived small RNAs. The small RNAs derived from CVB3 had: (i) a broad size distribution, (ii) a strong bias for positive strands over negative strands and (iii) covered the entire viral genome. Furthermore, replication of CVB3 with the D24A 3A substitution was not improved in AGO-2 knockout cells (which lacked a functional siRNA-mediated RNAi response), which argues against the ability of CVB3 3A to suppress RNAi.

## 12. The Viral Replication Complex and vRNA Replication

Replication of picornavirus vRNA takes place within a RC. The RC forms in close association with membranes in a highly controlled process that involves vRNA translation and viral protein processing, and multiple host proteins [209,210]. The P3 proteins (3A, 3B, 3C^pro^, 3D^pol^) and their processing intermediates (e.g., 3AB and 3CD^pro^) are the main viral proteins involved in forming the RC and vRNA replication. In line with this, substitutions in the cytoplasmic domain of PV 3A (which will also be present in 3AB) are lethal and reduced vRNA synthesis [98]. Below, we focus on the roles of 3A/3AB in the RC, and the roles of other viral nsps are only described briefly and only when relevant to the functions of 3A and 3AB.

The 3AB and 3CD^pro^ precursors of PV are relatively stable in infected cells (see above) and interact to form a complex with the CL at the 5’ end of positive-sense vRNA that nucleates formation of the RC [15,16,119,211,212,213]. The interaction of 3AB with the CL is dependent on 3CD^pro^, as 3AB does not bind to the CL alone [211]. The complex of 3AB and 3CD^pro^ can also bind to the 3’-end of plus strand vRNA [211,214]. However, in contrast to the binding to the CL, 3AB can bind to the 3’-end of PV RNA in the absence of 3CD^pro^ [211]. A more recent study [215] showed that the initial interaction with the CL is most likely mediated by the full length P3, which presumably supplies the copies of 3AB and 3CD^pro^ that form the complex. Additional molecules of P3 are subsequently recruited to the RC, which provides 3D^pol^ and VPg (3B) for vRNA replication. These observations are consistent with earlier studies that showed that PV RNA with changes in either 3A or 3B (VPg), that rendered viruses replication-defective, could only be rescued *in trans* by expression of full-length P3, and not by 3AB or other 3A- and 3B-containing precursors [141,216]. Similarly, defective replication due to a lesion in 3A of an FMDV replicon could be rescued by a “helper” replicon expressing wt 3A when supplied as part of the complete P3 [217].

The 3AB precursor is a membrane binding protein and it is conceivable that P3 is tethered to cellular membranes during formation of the RC by the MBR of 3A. As described above, this is most likely mediated by binding only on the cytoplasmic face of the membrane, as this would keep the P3 proteins on the same side of the membrane to facilitate polyprotein processing and formation of the RC. In addition, the ability of 3AB to bind cell membranes is important for vRNA replication as certain substitutions for the Met at position 79 in the MBR of PV 3A resulted in a specific defect in positive-strand vRNA synthesis (note: VPg uridylylation and negative-strand RNA synthesis occurred normally) [140]. However, this defect was not caused by an inability of 3A to bind membranes, as membrane association of 3A still occurred despite the changes [218]. Other substitutions within the HD of PV 3A cause aberrant processing of precursor proteins and result in non-viable viruses [138]. Together, these observations suggest that a precise interaction of 3A/3AB with membranes is required for proper formation and functioning of the PV RC. In line with this, when membrane associated, 3AB binds to 3D^pol^ and this interaction is believed to anchor 3D^pol^ to the RC [140,219]. Furthermore, 3AB has been shown to stabilize a complex between an RNA template, primer and 3D^pol^ to stimulate the RNA polymerase activity of 3D^pol^ [98,214,220,221,222,223]. In contrast to 3AB, 3A alone cannot bind or stimulate the polymerase activity of 3D^pol^ [214,224,225]. This suggested that the 3D^pol^ binding and stimulation activity of 3AB resides mainly within the 3B region. However, although 3B was shown to enhance 3D^pol^ activity, it did so with a reduced efficiency (on a molar basis) compared to 3AB [214,221], which suggests that the 3A and 3B regions of 3AB may have a synergistic effect on 3D^pol^ activity.

## 13. Uridylylation of VPg (3B)

3AB has been proposed to serve as the VPg donor for uridylylation. VPg is encoded by the 3B region of the genome and serves as the primer for vRNA synthesis [13]. To function as a primer, VPg is first uridylylated on a conserved Tyr residue at position 3. VPg uridylylation can be achieved in vitro using the VPg peptide with either poly(A), or the viral *cre* as the template [50]. It is not known if “free” VPg or a larger VPg-containing precursor (e.g., 3AB or 3BC) serves as the substrate for VPg uridylylation in vivo. For PV, the 3AB precursor protein was initially thought to serve as the donor for VPg uridylylation as it is present in relatively high amounts in infected cells [226,227]. However, the relatively high abundance of 3AB could reflect that it is not used for uridylylation, as this would necessarily be followed by 3AB processing to 3A and VPg-linked vRNA. For PV, some studies have concluded that a free N-terminus of VPg is required for uridylylation [94,216], which also suggests that 3AB is not the preferred substrate. Consistent with these observations, neither soluble nor membrane bound 3AB was shown to be a substrate for VPg uridylylation with either a poly(A) or a *cre* template [94,216,221].

A number of other observations point towards 3BC serving as the substrate for VPg uridylylation. This includes (i) PV 3BC can serve as a substrate for uridylylation and is more efficiently uridylylated than the “free” VPg peptide [100,216], (ii) preventing cleavage between 3A and 3B is lethal for PV replication [216], whereas preventing cleavage between 3B and 3C is not [100], and (iii) PV replicons produced vRNA covalently attached to 3BC if cleavage between 3B and 3C is prevented [100]. Similar observations were made for EMCV where 3BC was also linked to vRNA in recombinant viruses where processing of the 3B/3C cleavage site was impaired [228]. However, for FMDV, although the 3B-containing precursors 3B_1_B_2_B_3_C and 3B_3_C could be uridylylated in vitro, free 3B_3_ was uridylylated much more efficiently on an equimolar basis, than either of the larger 3B precursors [229]. Whether this difference for FMDV is a consequence of having three copies of VPg is unknown, but it suggests that the mechanism of VPg uridylylation for FMDV may differ from that of the enteroviruses.

## 14. Formation of Replication Organelles

During the initial phase of infection, it is likely that the RC assembles on pre-existing membranes of the early secretory pathway [230,231,232,233,234,235,236,237]. However, as infection proceeds, RCs assemble in association with newly formed membranes to generate structures that have been termed RO. Replication organelles are thought to increase the efficiency of vRNA replication by increasing the local concentrations and maintaining the correct spatial associations of viral and cellular proteins and vRNA within the RC [233,238]. Current evidence suggests that RO are novel membrane contact sites (MCS) (MCS are micro-domains formed by close contacts between cellular organelles that allow lipid exchange between apposed membranes) that form between the RC and cellular organelles. Several host proteins are involved in forming RO. Most of these proteins normally function in vesicular membrane traffic and/or non-vesicular lipid-transfer and are recruited to RO to drive the lipid exchange reactions that give RO their specific lipid identity. The main host proteins implicated in forming RO are GBF1, ARF1, ACBD3, OSBP (Oxysterol-binding protein), PI4KB and PI4KIIIα (from here onwards termed PI4KA). Detailed description of the functions of host proteins in RO is beyond the scope of this review; however, before describing how 3A interacts with host proteins to generate RO, it seems appropriate to summarize how host proteins contribute to their formation. RO have been mostly studied using enteroviruses, kobuviruses and cardioviruses; therefore, these viruses will be used as examples.

**Figure 4:**
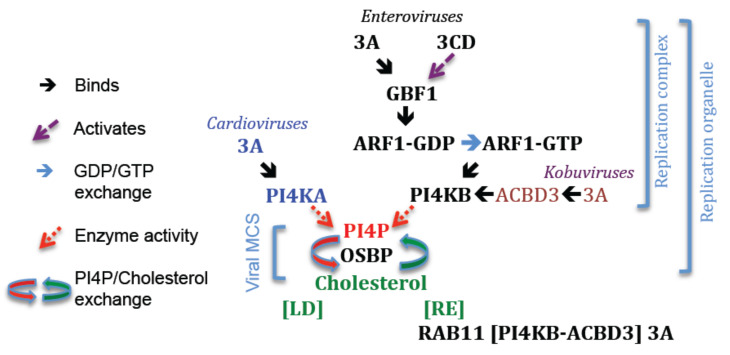
Key steps in forming RO enriched for PI4P and cholesterol. Cardioviruses; PI4KA is recruited to RO by a direct interaction with 3A. Kobuviruses; PI4KB is recruited to RO when in a complex with ACBD3 and 3A. Enteroviruses; How PI4KB is recruited to RO is unclear. The roles of 3A, 3CD and GBF1 in recruiting PI4KB to RO are discussed in the main text. At RO, PI4P recruits OSBP, which then drives exchange of PI4P for cholesterol. Cholesterol may be supplied by lipid droplets (LD) or derived from endocytosed cholesterol. For the enteroviruses this involves 3A (at Table 1. at recycling endosomes (RE) and may involve PI4KB and ACBD3. GBF1, Golgi brefeldin A–resistant guanine nucleotide exchange factor 1; ARF1, ADP-ribosylation factor 1; PI4KA, Phosphatidylinositol 4-Kinase IIIα; PI4KB, Phosphatidylinositol 4-Kinase IIIβ; PI4P, Phosphatidylinositol 4-phosphate; OSBP, Oxysterol-binding protein; MCS, membrane contact site.

The membranes of RO formed in cells infected by enteroviruses, kobuviruses and cardioviruses are enriched in phosphatidylinositol 4-phosphate (PI4P) and cholesterol [231,232,234,239,240]. The function(s) of PI4P and cholesterol in the RO are unclear. However, both are needed for efficient vRNA replication and appear to modulate viral polyprotein processing [233,239,241]. In addition, PI4P has been proposed to provide a binding-surface for 3D^pol^ and/or 3CD^pro^ [232,242]. Membrane levels of cholesterol are normally maintained by OSBP [243], which mediates exchange of PI4P for cholesterol between apposed membranes at MCS [244]. In infected cells, OSBP is redirected to RO where it serves the same function and exchanges PI4P for cholesterol [234,245,246], and this is thought to account for the accumulation of most of the cholesterol at RO. Cholesterol is an important component of cellular membranes and regulates fluidity, thickness, and intrinsic curvature of lipid bilayers. The p33 protein of Tomato bushy stunt virus is a membrane binding protein and interacts with cholesterol via CRAC (Cholesterol recognition/interaction amino acid consensus) and CARC (an inverted CRAC) cholesterol-binding motifs that lie within, or adjacent to, its transmembrane domain [247]. Interestingly the HD of PV 3A contains a short sequence 72-**Val**-Ala-Gly-**Val**-Val-**Tyr**-Val-Met-Tyr-**Lys**-81 (the CRAC residues are in bold) that lies within the MBR identified experimentally [94] and shows similarity to a CRAC motif ([Leu/**Val**]-X^1–5^-[**Tyr**]-X^1–5^-[**Lys**/Arg], where X represents apolar residues compatible with the hydrophobic membrane environment). However, the presence of a CRAC motif does not always indicate cholesterol binding and the ability of PV 3A to bind cholesterol will require experimental validation. High levels of PI4P are maintained in RO by type III PI4K. Despite sharing the need for PI4P for replication, different picornaviruses commandeer different PI4K enzymes for this purpose (Figure 4). Enteroviruses and kobuviruses recruit PI4KB, whereas cardioviruses recruit PI4KA to their RO. How PI4K is redirected to RO has been the subject of many studies and is the main focus of the remainder of this review. However, it should be noted that certain other picornaviruses, such as HAV and FMDV, replicate independently of PI4K (PI4KA and PI4KB), and PI4P is not enriched within RO of FMDV-infected cells [248,249]. Thus, it is likely that HAV and FMDV (and some other picornaviruses) have different requirements for host proteins and generate RO by different mechanisms.

## 15. Recruitment of PI4K to Replication Organelles

In cardiovirus- and enterovirus/kobuvirus-infected cells, PI4P is generated in RO by PI4KA and PI4KB, respectively [231,232,234,239,240,250,251]. PI4KA and PI4KB are soluble cytosolic proteins that are normally recruited to cell membranes (PI4KA; to the ER, early Golgi and the plasma membrane, PI4KB; primarily to the Golgi) by direct interactions with specific compartment resident-proteins [252,253,254,255,256,257,258,259]. In addition, PI4KB can be recruited to the Golgi by ARF1 activation, although in this case the cellular protein that tethers PI4KB to the Golgi has not been identified [260]. The 3A proteins of cardioviruses provide a similar function to cellular tethering-proteins and recruit PI4KA to RO via a direct interaction [231]. In immunoprecipitation experiments, the 3A proteins of cardioviruses were shown to interact with PI4KA, and in cells co-transfected with PI4KA and either 3A or 3AB, PI4KA was co-localized at 3A/3AB positive sites [231]. This suggests that either cardiovirus 3A or 3AB could serve to directly anchor PI4KA in RO. In contrast to cardioviruses, recruitment of PI4KB to enterovirus/kobuvirus RO requires cellular proteins that normally tether PI4KB to the Golgi. Interestingly, although enteroviruses and kobuviruses recruit PI4KB to RO they appear to use different mechanisms (discussed below). For the kobuviruses, 3A has a central role in recruiting PI4KB to RO, whereas for the enteroviruses the role of 3A is less clear, and it is possible that recruitment occurs by both 3A-dependent and 3A-independent processes. The roles of 3A and cellular proteins in recruiting PI4KB to RO are described in more detail below.

## 16. Recruitment of PI4KB by Subversion of GBF1/ARF1

### 16.1. The Case for 3A

The study by Hsu et al. [232], which first showed that PI4P and PI4KB are present in RO and are required for enterovirus replication was a major advance in understanding how RO form and function. Moreover, this study has since underpinned numerous other studies that have confirmed PI4KA and PI4KB as important host proteins for picornavirus replication. Indeed, for many picornaviruses, PI4P is now considered a marker for the RC/RO [231,232,234,239,240,251]. As well as PI4KB, GBF1 and ARF1 are also present in enterovirus RO [232]. In addition, GBF1, ARF1 and PI4KB were shown to co-localize at membrane sites labelling positive for enterovirus 3A in 3A-transfected cells [232]. As the 3A proteins of PV and CVB3 are known to interact directly with GBF1, these observations suggested that 3A could recruit PI4KB to RO by subverting the normal cellular pathways (e.g., membrane recruitment of PI4KB by GBF1 induced activation of ARF1) that recruit PI4KB to the Golgi. Interestingly, although PI4KB was present within enterovirus RO, and at sites of 3A-expression in 3A-transfected cells, the COPI coatomer (which binds to activated ARF1) was absent [232]. Observations made earlier also showed that expression of CVB3 3A appeared to inhibit membrane recruitment of the COPI coatomer [95,128]. To explain these observations, it was suggested that the interaction between activated ARF1 and the COPI coatomer could be prevented in RO to favour ARF1 recruitment of PI4KB [232].

Before PI4KB was shown to be important for enterovirus replication, there was considerable evidence that GBF1 was also involved. GBF1 became the focus of studies on enterovirus replication primarily because of the observation that BFA inhibited PV replication [261,262]. At that time, BFA was known to inhibit ARF1 activation (by preventing GDP/GTP exchange) and induce profound morphological changes in membranes of the secretory pathway [263,264,265]. Thus, the sensitivity of PV to BFA suggested that membrane or protein components of the secretory pathway (such as GBF1 and ARF proteins) could be required for PV replication. More direct evidence for the requirement for GBF1 in enterovirus replication came from studies which showed that: (i) PV and CVB3 replication can be rescued from BFA-inhibition by overexpression of GBF1, but not by GBF1 lacking GEF activity, and (ii) the ability to overcome BFA-inhibition was improved by including substitutions in the GBF1 catalytic domain that resulted in BFA-resistance [266,267]. Furthermore, enterovirus replication is inhibited by siRNA knockdown of GBF1 [266,267]. GBF1 normally cycles between membrane-bound and cytosolic states and preventing its catalytic activity prolongs its interaction with membranes [132,134]. Thus, it was suggested that the failure of catalytically inactive GBF1 to rescue PV replication could result from increased membrane association of GBF1, rather than from a lack of GEF activity [268]. However, a GBF1 mutant with normal membrane cycling dynamics, but an impaired ability to activate ARF1 was unable to rescue PV replication [268], which confirmed that the GEF activity of GBF1 is required for enterovirus replication.

In line with the requirement for GBF1, a number of observations have suggested that ARF proteins are also required for enterovirus replication. A role for ARF proteins was suggested by early studies that reported that synthetic peptides with sequences corresponding to the N-terminal residues of ARF1 could inhibit cell-free replication of PV [261]. More recent studies have shown that the amount of activated ARF1-GTP gradually increases as infection progresses, and that ARF1 locates to the RC in PV-infected cells [232,268,269]. However, it should be noted that other observations have suggested that ARF proteins may not be involved in enterovirus replication. These include: (i) knockdown of ARF1 does not significantly inhibit infection by enteroviruses [232,267,270], and (ii) overexpression of a constitutively active mutant of ARF1 (Q71L-ARF1), when expressed alone or when co-expressed with other constitutively active ARF mutants (ARF3, ARF4 or ARF5), does not rescue enterovirus replication from BFA-inhibition [266,267,271]. Collectively the above studies confirmed that catalytically active GBF1 is required for enterovirus replication and supported a model in which recruitment of PI4KB to enterovirus RO is initiated by 3A, and most likely dependent on activation of ARF1. However, as described above (Section 6), some enterovirus 3A proteins are thought to inhibit the GEF activity of GBF1 (and therefore ARF1 activation) and this provides a mechanism to inhibit the secretory pathway [95,128]. Thus, the role of enterovirus 3A in inhibiting GBF1 and the secretory pathway appears at odds with the mechanisms proposed for 3A-mediated recruitment of GBF1, ARF1 and PI4KB to RO.

The role of enterovirus 3A in recruiting PI4KB to RO has also been challenged by other evidence that suggests the existence of a 3A-independent mechanism(s) for recruiting PI4KB to RO. This comes primarily from studies that used 3A proteins with a serine-insertion after residue 15 (called 3A-ins 16S, in CVB3; and 3A-2, in PV 3A), which no longer bind GBF1 and are incapable of inhibiting protein secretion. One such study showed that membrane association of ARF proteins was increased to similar levels on translation of full-length PV RNAs with either the wt 3A sequence or with the 3A-2 mutation [272]. This suggested that 3A may not have a direct role in recruiting GBF1/ARF1, and therefore PI4KB to RO [268]. Moreover, despite rendering 3A incapable of binding to GBF1 [96], viruses (and sub-genomic replicons) with the 3A-2 (PV) or 3A-ins 16S (CVB3) substitutions are viable [96,123], which shows that 3A binding to GBF1 is not required for enterovirus replication. Furthermore, these viruses remained sensitive to BFA and could be rescued from BFA-inhibition by overexpression of GBF1 [267,268]. Similar to PV with the 3A-2 change, recombinant PVs [113] with an epitope (myc or FLAG) tag inserted into the N-terminal region of 3A are also viable despite the tags preventing 3A binding to GBF1. Furthermore, the 3A-tagged viruses were shown to be sensitive to BFA and could be rescued from BFA-inhibition by expression of GBF1. However, although enteroviruses with 3A-ins 16S, 3A-2 or epitope tags in 3A are viable they show a slightly delayed and/or a reduced level of replication [113,266,268]. It was suggested that these characteristics could be explained if the serine insertions/tags weaken, rather that completely block 3A binding to GBF1 [113,266]. This could also explain how GBF1 can rescue replication of viruses (and replicons) carrying such mutations from BFA. However, the serine/tag insertions will also be present in the 3AB precursor, which is essential for vRNA replication and formation of the RC [98]. Thus, it is possible that introducing changes into 3A could reduce vRNA replication by influencing the interactions of 3A and/or 3AB with other viral or cellular proteins within the RC.

Additional evidence that supports the existence of a 3A-independent mechanism for recruitment of PI4KB to enterovirus RO comes from studies with RV-A2. The 3A protein of RV-A2 does not bind to GBF1 [273]. However, RV-A2 replication requires PI4KB [234] and is sensitive to BFA [273,274] and can be rescued from BFA-inhibition by overexpression of a BFA-resistant GBF1 [273]. Moreover, GBF1 locates to RO in RV-A2-infected cells [273]. Collectively, these observations suggest that RV-A2 replication is dependent on GBF1 despite the inability of 3A to bind to GBF1 [273].

Consistent with the model in which 3A binding to GBF1 triggers recruitment of PI4KB to RO, Belov et al. [266] showed that translation of PV replicon RNA with a wt 3A sequence caused an increased membrane association of GBF1. They also showed that translation of PV RNA with the 3A-2 modification also increased the amount of membrane-associated GBF1; however, to a lesser extent than for the replicon RNA with a wt 3A sequence. This suggested that 3A-2 could either bind to GBF1 with a lower affinity than wt 3A, or that GBF1 was recruited to membranes by a different viral protein. In addition to enhancing GBF1 membrane binding, translation of wt PV RNA also induced membrane association of ARF1; however, in contrast to other studies that showed that enterovirus 3A prevents membrane association of COPI, they also observed increased membrane association of α-COP (a component of the COPI coatomer) [266]. This suggested that 3A binding to GBF1 could trigger ARF1 activation and membrane recruitment of COPI. These observations appear to contradict the following: (i) the model proposed by Hsu et al. [232] in which 3A-mediated recruitment of GBF1/ARF1 to RO leads to the preferential recruitment of PI4KB over COPI, and (ii) the proposed role for 3A in inhibiting protein secretion [95], i.e., 3A binds to GBF1 and inhibits its GEF activity, and hence, ARF1 activation and COPI recruitment. The reasons why COPI membrane association appears to decrease [95,128,232] or increase [266,275] on expression of enterovirus 3A are currently unknown but could be the result of the different experimental approaches. To reconcile their results with the ability of 3A to inhibit protein secretion, it has been suggested that GBF1 may provide a function when in the RC that differs to it normal cellular functions [276], or that 3A could inhibit protein secretion indirectly by sequestering GBF1 at the RC [266], and thereby reduce the amount available for the secretory pathway. However, an alternative explanation was proposed by Altan-Bonnet and Balla [277] who suggested that 3A may inhibit formation of COPI-coated vesicles at a late stage of formation. During COPI vesicle formation, the preformed COPI coatomer interacts with activated ARF1 (ARF-GTP). However, before it engages ARF1-GTP, the COPI coatomer transiently associates with GBF1 and this occurs before GDP/GTP exchange and ARF1 activation [278]. Thus, it is interesting to speculate that 3A binding to GBF1 stabilizes a complex between GBF1 and ARF1-GDP, which cannot proceed to GDP/GTP exchange but can still interact with COPI coatomer. A similar study also showed that expression of CVB3 3A induced membrane association of GBF1, ARF1 and αCOP [275]. In addition, this study showed that expression of CVB3 3A also induced increased membrane association of PI4KB, which also suggested that 3A binding to GBF1 could trigger ARF1 activation. However, subsequent studies have shown that enterovirus 3A can recruit PI4KB to RO when in a complex with ACBD3, which normally tethers PI4KB to Golgi membranes (see below); thus, the ability of 3A to bind to ACBD3 could account for the increased membrane association of PI4KB induced by 3A [275].

Interestingly, more recent studies have shown that expression of PV 3A (or 3AB) in isolation reduced PI4P levels [242,245]. These observations were unexpected and also suggest that 3A may not be involved in recruiting functionally active PI4KB to RO and are discussed in more detail below. In retrospect, although the study by Hsu et al. [232] showed that GBF1, ARF1 and PI4KB were recruited to the same membrane sites as 3A, the level of PI4P in 3A-transfected cells was not determined, and a direct link between 3A and enrichment of PI4P at RO was not demonstrated.

### 16.2. The Case for 3CD^pro^

Most studies investigating the mechanism of PI4KB recruitment to enterovirus RO have focused on 3A. However, PV 3CD^pro^ has also been implicated in activating the GBF1/ARF1/PI4KB pathway. Studies by Belov et al. [272,279] showed that expression of PV 3CD^pro^ increase membrane association of activated ARF proteins. More recently, expression of PV 3CD^pro^ was shown to induce membrane proliferation and PI4P synthesis [242]. The increased level of PI4P induced by 3CD^pro^ appeared to require GBF1, ARF1 and PI4KB as it could be inhibited by BFA (or by golgicide A) or PIK93 (an inhibitor of PI4KB), and was accompanied by an increased level of ARF1-GTP (i.e., ARF1 activation). Interestingly, whereas 3A specifically induced membrane association of GBF1, the expression of PV 3CD^pro^ preferentially increased membrane association of two other ARF GEFs, namely BIG1 (brefeldin A-inhibited guanine nucleotide-exchange factor 1) and BIG2 and did not induce membrane association of GBF1 [269]. These observations suggested that BIG1/2 could be involved in enterovirus replication; however, a number of other studies have concluded that neither BIG1 nor BIG2 (BIG1/2) are involved in this process [95,266,267,280]. A recent study has identified a functional link between GBF1 and BIG1/2 [281]. This showed that activation of GBF1 enhanced membrane recruitment of BIG1/2 by a process that involved the GBF1-activated ARF proteins, ARF4 and ARF5. This suggests the possibility that the increased membrane association of BIG1 and BIG2 induced by 3CD^pro^ could be a consequence of increased activity of GBF1, rather than reflecting the need for BIG1 and BIG2 for enterovirus replication. Consistent with this possibility, a recent study has shown that ARF4 and ARF5 are located within enterovirus RO [282].

The study by Banerjee et al. [242] also showed that when expressed alone PV 3A or 3AB reduced the amount of PI4P in cells. This observation is consistent with an earlier study that also concluded that expression of PV 3A (and to a lesser extent 3AB) reduced levels of PI4P [245]. These observations suggest that PV 3A does not activate (or indirectly facilitate activation of) PI4KB. Interestingly, the reduction in PI4P caused by 3AB could be overcome by co-expression with 3CD^pro^ [242]. As the authors point out, this was not unexpected as PI4P levels are increased in PV infected cells [283], thus the positive effect of 3CD^pro^ on PI4P synthesis would be expected to dominate over the inhibitory effect of 3AB. Furthermore, 3AB and 3CD^pro^ are relatively abundant in PV-infected cells and form a complex with the 5’-CL and it is possible that 3AB, when bound to 3CD^pro^, may no longer inhibit PI4P synthesis. In contrast, the inhibitory effect of 3A on PI4P levels was not overcome by co-expression with 3CD^pro^. In PV-infected cells, 3A is produced by the minor P3 processing pathway and is present at relatively low levels compared to 3CD^pro^, especially during the early phase of infection [99,100,284]. Thus, it is possible that during a natural infection the inhibitory effect of 3A on PI4P levels may be outweighed by the ability of 3CD^pro^ to induce PI4P synthesis, especially during the early phase of infection when the levels of 3A are low.

## 17. 3A and the Great Escapes

As discussed above, GBF1 is required for replication of enteroviruses, which can therefore be inhibited by BFA. Studies using expression of viral nsps in isolation have shown that 3CD^pro^ (but not 3A) increased levels of PI4P and this could also be inhibited by BFA [241,244,307} This suggests that 3CD^pro^ and GBF1 are important parts of the pathway that normally generates PI4P at RO [242]. However, PVs with resistance to BFA have been isolated but they did not have aa changes in 3CD^pro^ but instead had single aa substitutions in 2C (Val to Ile at residue 80) or 3A (Ala to Val at residue 27) [285]. PVs with either change were able to replicate in the presence of BFA, but replication was improved further for viruses with both changes. The molecular mechanisms that underlie how changes in 2C and/or 3A can overcome inhibition of PV replication by BFA are currently unclear. A study by Arita [245] reported that PV P2 proteins interacted with PI4KB in an M-2-H assay (2B, 2C and 2BC) and a proximity ligation assay (2BC), and that cells transfected to express 2BC had increased levels of PI4P. However, 2BC was not immunoprecipitated with FLAG-tagged PI4KB, and the increased level of PI4P in 2BC-transfected cells was not prevented by inhibition of PI4KB. Nevertheless, these observations suggest that 2C/2BC may interact with PI4KB and that the aa changes seen in the 2C of BFA-resistant PV isolates could potentially allow for more efficient recruitment of PI4KB to the RC in the presence of BFA.

Additionally, as discussed above, replication of several picornaviruses is dependent on PI4KB and OSBP, and thus inhibitors of PI4KB and OSBP also restrict their replication [234,286,287,288,289,290,291,292]. Enteroviruses with resistance to inhibitors of PI4KB and OSBP have been isolated and carry single aa changes in 3A. CVB3 isolates that are resistant to PI4KB inhibitors have Val-45-Ala, Ile-54-Phe or His-57-Tyr substitutions whereas PVs that are resistant to PI4KB inhibitors have an Ala-70-Thr change [287,288,293,294]. These changes in CVB3 3A did not restore PI4KB activity but allow for replication independently of PI4KB [287]. The same aa changes in CVB3 3A were seen in virus isolates that were selected to be resistant to OSBP inhibitors [289,292], which highlights the strong link between PI4KB and OSBP during enterovirus replication. Recently, the mechanism by which the His-57-Tyr change in 3A overcomes inhibitors of PI4KB and OSBP has been identified [295]. Using a replication-independent expression system, both types of inhibitors were shown to cause defective processing of wt 3AB, and that the His-57-Tyr change in 3A corrected this defect and restored normal 3AB processing. These results show that inhibition of either PI4KB or OSBP impairs processing at the boundary between 3A and 3B and suggest that correct 3AB processing is dependent on cholesterol. In line with these observations, Arita [291] showed that the Ala-70-Thr change in PV 3A, that results in resistance to PI4KB inhibitors, increased the level of 3A, and decreased the level of 3AB during infection. In addition, other aa changes in 3A (Thr-14-Met and His-86-Tyr) have been identified in PVs that are resistant to PI4KB inhibitors. When combined, these changes confer similar resistance to PI4KB inhibitors as the Ala-70-Thr change and also enhance the level of 3A. This suggested that restoring aberrant 3AB processing in the presence of PI4KB inhibitors (and possibly OSBP inhibitors) could be a common mechanism used by enteroviruses to overcome inhibition of PI4KB. A more recent study, by Arita and Bigay [296], showed that aa changes (Glu-53-Asp and Arg-54-Trp) in 3A allowed for PV replication in PI4KB^ko^ cells. Similar to the aa changes in 3A in PI4KB-resistant CVB3 isolates, these changes also allowed PV replication in the presence of an OSBP inhibitor. In addition, these changes appeared to enhance processing of 3AB.

EMCV requires PI4KA for replication [231]. EMCV isolates that are less dependent on PI4KA have been isolated using cells that expressed a low level of PI4KA and support limited replication of wt EMCV [297]. Viruses that showed improved growth in these cells were less sensitive to inhibition of PI4KA (by AL-9) and also carried a single aa change (Ala to Val) at either residue 32 or 34 in 3A. Viruses with these changes in 3A do not require other PI4K isoforms (PI4K2A, PI4K2B or PI4KB) and their replication was independent of PI4P. In RO, PI4P serves to recruit OSBP and cholesterol, however, when cells were infected with the mutant viruses (with the changes in 3A) in the presence of AL-9, co-localization of 3A with OSBP and cholesterol was greatly reduced [297]. This suggests that EMCV isolates with changes in 3A do not require high levels of cholesterol for infection. Interestingly, unlike CVB3 where the same aa changes in 3A result in resistance to both PI4KB and OSBP inhibition [287,288,289,290]. EMCV with changes in 3A that allowed for PI4KA independent replication remained sensitive to OSBP inhibitors. These observations show that replication of EMCV with the above changes in 3A remain dependent on OSBP and suggest that OSBP may provide another non-canonical function during EMCV replication [297].

## 18. Recruitment of PI4KB by Subversion of ACBD3

Similar to the enteroviruses, AiV replication is dependent on PI4KB and generates RO enriched with PI4P and cholesterol [258]. However, AiV is insensitive to BFA and does not require GBF1 for replication [258], which suggests that AiV recruits PI4KB to RO by a GBF1/ARF1-independent mechanism. PI4KB can be recruited to the Golgi via interactions with several different Golgi-resident proteins [254,255,256,259,298,299], including ACBD3, which interacts directly with PI4KB [258,300]. In 2012, two independent studies showed that ACBD3 relocates to AiV RO and is required for virus replication [89,258]. In addition, these studies showed that AiV 3A binds to ACBD3 [89,258]. Furthermore, although AiV 3A did not directly interact with PI4KB it was co-purified along with ACBD3 when using strep-tagged 3A as bait [89,258]. This indicated that ACBD3 could simultaneously bind to AiV 3A and PI4KB, and that this could facilitate delivery of PI4KB to RO [89,258]. Interestingly, substitutions in 3A were identified that prevented co-purification of PI4KB without appearing to affect the interaction with ACBD3, which suggested the possibility that AiV 3A could make weak or transient interactions with PI4KB that are stabilized by ACBD3 [89]. The ACBD3 protein consists of two central domains (a charged amino acid region, termed the CAR domain, and a glutamine-rich domain, termed the Q-domain) that are flanked by an N-terminal acyl-CoA-binding domain and a C-terminal Golgi Dynamics (GOLD) domain. The binding sites for 3A and PI4KB have been mapped to the GOLD- and Q-domains of ACBD3, respectively [89,258], which explains how ACBD3 can simultaneously bind to 3A and PI4KB. ACBD3 lacks intrinsic membrane binding capability and is targeted to Golgi membranes through an interaction between the GOLD domain and giantin, which is a member of the Golgin family of proteins that regulate Golgi architecture and function [301,302]. However, giantin is not present in AiV RO, which suggests that 3A can displace giantin from the GOLD domain and that this may facilitate recruitment of ACBD3 to RO.

In addition to identifying ACBD3 as a binding partner of AiV 3A, the study by Greninger et al. [89] (and the subsequent study by Greninger et al. [303]) also showed that PV 3A could interact with ACBD3. Furthermore, these studies suggested that ACBD3 was required for PV replication, as replication was inhibited by ACBD3 knockdown. Following these observations, other studies gave conflicting results, as knockdown of ACBD3 was reported to enhance PV infection [304] and did not appear to influence replication of CVB3 or RVs [273,275]. However, follow-up studies have shown conclusively that ACBD3 is essential for enterovirus replication, as replication of EV-A71 and CVB3 was severely impaired in ACBD3 knockout (ACBD3^KO^) cells and could be restored by exogenous expression of ACBD3 [251,305,306]. In addition, 3A, ACBD3 and PI4KB were located at EV-A71 RO [251]. A more recent study, that also used ACBD3^KO^ cells, confirmed that ACBD3 is required for replication of a number of human enteroviruses (EV-A71, CVB3, PV, EV-D68, RV-A2 and RV-B14) [307]. Furthermore, in 3A-transfected cells, enterovirus 3A was co-localized with ACBD3 and PI4KB, whereas in 3A-transfected ACBD3^KO^ cells no co-localization with PI4KB was observed. These observations are similar to those made for AiV 3A and suggest that PI4KB may be recruited to RO by enterovirus 3A when in a complex with ACBD3 [307]. However, Horova et al. [107] showed that when expressed in isolation, EV-D68 3A does not increase levels of PI4P. Based on these observations, it was proposed that for enteroviruses either another viral protein(s) is required to co-operate with 3A to increase PI4KB activity during infection [107] or, alternatively, that ACBD3 could provide a scaffold function for the correct positioning (and function) of 3A in the RC/RO, rather than serving to recruit active PI4KB [307].

The crystal structures of the N-terminal domains (i.e., 3A truncated at the HD) of the AiV-A and AiV-B 3A proteins have been determined when in a complex with the ACBD3 GOLD domain. This showed that these 3A proteins adopt a similar overall structure when bound to the GOLD domain despite sharing a low sequence similarity [308] (see Figure 1). In the complex, the 3A N-terminal domain adopts an ordered conformation and wraps around the GOLD domain so that the N- and C-termini of the N-terminal domain make contacts on opposite sides [308]. Should full-length 3A bind to the GOLD domain in the same way, then this configuration would allow the N-terminal myristate group and the MBR of the AiV 3A to simultaneously engage with a single bilayer and thereby effectively “staple” ACBD3 to the membrane. Crystal structures have also been solved for the 3A proteins from enteroviruses (PV, EV-A71, EV-D68, and RV-B14) in complex with the GOLD domain [107] (Figure 3). The overall structures of these complexes were highly similar to each other despite wide aa sequence variation across the 3A proteins. Electron density could not be assigned for the first 15 N-terminal residues of the crystalized enterovirus 3A proteins, which suggests that this region is flexible and does not adopt a stable conformation when 3A is bound to ACBD3. This is different to the AiV 3A:GOLD complex where residues close to the N-terminus of 3A make contacts with the GOLD domain. The N-terminal residues of enterovirus 3A (but not AiV 3A) include the binding site for GBF1 and it is likely that the above difference reflects the need for some enterovirus 3A proteins to interact with GBF1 [107]. The rest of the enterovirus 3A polypeptide formed four distinct structural elements (two α helices and two β strands) that were conserved in all four structures and each contributed to interaction with the GOLD domain. Mutagenesis studies confirmed that all four secondary elements are important for binding to ACBD3 and for enterovirus replication. Consistent with the ability of PV 3A to form homodimers [104], 3A made contacts with a second 3A molecule when bound to ACBD3. In agreement with the NMR structure, the 3A dimerization interface was mainly formed by hydrophobic interactions between residues of the central α-helices, and Ala substitution of the main residues (Leu25, Val29, Val34, and Tyr37) involved in forming the interface impaired dimerization and also replication of EV-D68 [107]. In the NMR structure, the N-terminal domain of PV 3A appeared largely unstructured apart from the two central α helices [104]. However, in the EV-D68 3A:GOLD complex, the C-terminal residues of the N-terminal domain adopted an ordered conformation. This suggested that this region could become ordered on binding to the GOLD domain. However, it is also possible that this region of enterovirus 3A is normally ordered within the context of full-length 3A.

A comparison of the AiV and enterovirus 3A:GOLD complexes showed that despite having diverse sequences and distinct secondary structures, the 3A proteins of these viruses bind to the same regions of the GOLD domain [107]. Furthermore, the GOLD domain most likely interacts with membranes in a similar conformation as when in a complex with the different 3A proteins. ACBD3 serves as a scaffold for a number of protein complexes associated with the Golgi and the GOLD domain interacts with several different proteins [309,310]. Thus, the inherent ability of the GOLD domain to bind several diverse cellular proteins may account for the ability to interact with distinct 3A proteins of different picornaviruses [89]. However, although the 3A proteins of enteroviruses and kobuviruses interact with the same region of the GOLD domain, they interact in reverse orientations which suggests that the 3A proteins of different picornavirus genera have evolved the ability to interact with ACBD3 independently [107].

PI4P levels are increased in kobuvirus RO; however, it is not clear if simply recruiting PI4KB generates PI4P or if the kinase activity of the bound PI4KB is also up-regulated. Regulation of the enzymatic activity of PI4KB is not completely understood [298]. However, PI4KB activity is regulated (in part) through membrane recruitment. In an in vitro assay, the kinase activity of PI4KB was increased by ACBD3 but only when ACBD3 was anchored on artificial vesicles via a His-tag [300]. This suggested that ACBD3 does not directly influence the kinase activity of PI4KB but regulates its activity by positioning it on membranes in close proximity to its substrate [300]. Earlier studies had also reported that ACBD3 alone could not stimulate PI4KB kinase activity [240]. In addition, this study also reported that AiV 3A (and 3AB) alone does not stimulate PI4KB kinase activity. However, in the presence of both ACBD3 (at ≥5-fold molar excess relative to PI4KB) and 3A (at 25-fold molar excess relative to PI4KB), kinase activity was increased [240]. However, subsequent studies by McPhail et al. [311] concluded that AiV 3A can enhance the kinase activity of PI4KB in the absence of ACBD3 when 3A is membrane associated. This study used His-tagged AiV 3A (residues 1-59), which was anchored on the surfaces of artificial vesicles using DGS-NTA(Ni) (1,2-dioleoyl-sn-glycero-3-[(N-(5-amino-1-carboxypentyl)iminodiacetic acid) succinyl] (nickel salt)). When the assay was carried out on vesicles lacking DGS-NTA(Ni) neither ACBD3 nor 3A (when they were added either individually or together) induced kinase activity. However, when 3A was anchored to the DGS-NTA(Ni) vesicles, a dose-dependent activation of PI4KB was observed and this was enhanced when ACBD3 was included in the assay. These observations suggest that, when membrane bound, AiV 3A may be able to interact directly with PI4KB and stimulate its kinase activity, and this is further enhanced by ACBD3. Thus, ACBD3 may not only facilitate recruitment of PI4KB to RO by AiV 3A but may also provide a mechanism to accelerate PI4P synthesis at RO.

For AiV, a link between 3A expression and increased PI4P was demonstrated as PI4P levels were increased in 3A-transfected cells [258]. In contrast (as mentioned above), expression of enterovirus 3A in isolation either reduces or does not influence PI4P levels [242,245,307]. The reason(s) why the 3A proteins of AiV and PV affect levels of PI4P differently are currently unknown. As noted above, PV and AiV 3A bind to ACBD3 in opposite orientations and this could conceivably alter the interaction between ACBD3 and PI4KB in such a way as to differentially influence the enzyme activity of PI4KB, and hence PI4P levels. Other studies have also suggested that AiV and PV 3A proteins interact differently with ACBD3. The study by Greninger et al. [89] showed that ACBD3 and PI4KB could be readily co-purified along with strep-tagged AiV 3A in pull down experiments (under standard assay conditions), whereas only ACBD3 was co-purified when the assay was carried out using strep-tagged 3A of several enteroviruses. These results suggested that the interaction between ACBD3 and PI4KB may be stabilized by AiV 3A, but not by enterovirus 3A. Consistent with this conclusion, PI4KB was co-purified along with ACBD3 by enterovirus strep-tagged 3A proteins when the assay conditions were changed (to rapid-capture conditions) to better detect transient interactions. Differences in how AiV and PV 3A bind to ACBD3 have also been demonstrated using TCB1D22 (a putative Rab GAP) [312], which competes with PI4KB for overlapping binding sites on the Q-domain of ACBD3. In pull-down experiments using TBC1D22 tagged with V5 as bait, 3A was readily co-purified along with ACBD3 from cells transfected to express FLAG-tagged PV 3A [303]. However, when the experiment was repeated using cells transfected to express FLAG-tagged AiV 3A, although 3A was co-purified it was present at a greatly reduced level relative to ACBD3. These results suggested that TBCD1D22 could reduce the strength of the interaction between AiV 3A and ACBD3 [303]. Based on these observations, it was postulated that during infection, AiV 3A may displace TBC1D22 from ACBD3 to favour binding to PI4KB with a concomitant increased production of PI4P.

Finally, it has also been suggested that the apparent inability of enterovirus 3A proteins to stimulate the kinase activity of PI4KB when bound to ACBD3 results from the requirement for binding to GBF1 [107]. Interestingly, although PI4KB was not co-purified with ACBD3 in the experiments described above using enterovirus strep-tagged 3A (under the standard assay conditions), GBF1 was co-purified with it [89]. In these experiments, it was not possible to conclude if ACBD3 and GBF1 simultaneously bind to a single molecule of 3A, or if they were bound to separate 3A proteins. However, in the enterovirus 3A/GOLD domain complex the N-terminal residues of 3A that form the GBF1 binding site were apparently disordered, which suggests that this region could remain available to bind to GBF1 when 3A is bound to ACBD3 [107]. For RV-B14 3A, GBF1 was not pulled down with ACBD3 under the standard assay conditions but it was under rapid capture assay conditions [89]. This is in line with the observations that 3A of RV-B14 shows a reduced ability to bind to GBF1 (compared to the 3A proteins of PV and CVB3) [128]. This observation provides additional interest as 3A of RV-B14 has been shown not to inhibit protein trafficking through the secretory pathway [127,128].

As described above, enterovirus 3A proteins binds to ACBD3, yet expression of 3A alone does not increase PI4P levels [107, 307]. These observations suggested that ACBD3 could play a role in enterovirus replication independent of its ability to recruit and stimulate the kinase activity of PI4KB. Consistent with this, ACBD3 has been shown to bind to 3A proteins of other picornaviruses that do not require PI4KB for replication (e.g., HAV) [303]. This points to a role for ACBD3 in replication of some picornaviruses that is independent of the ability to bind to PI4KB. As noted above, cholesterol is important for picornavirus vRNA replication and polyprotein processing and is enriched in enterovirus RO by a process that involves cholesterol shuttling mediated by OSBP. However, other mechanisms are also thought to channel cholesterol to RO including the re-routing of endocytosed cholesterol from recycling endosomes (RE) [239]. In enterovirus-infected cells, RE are recruited to RO by a process facilitated by an interaction between PI4KB (at the RC/RO) and Rab11 (on RE) [239]. In cells transfected to express CVB3 3A, Rab11 was localized to sites where the 3A protein was present, which suggests that 3A is sufficient to target RE to the RC/RO [239]. Rab11 is an effector protein of PI4KB and binds to PI4KB independently of its kinase activity [255]. Similarly, the interaction between PI4KB and Rab11 in CVB3-infected cells does not require the kinase activity of PI4KB [239]. Thus, it was suggested that by binding to Rab11, PI4KB could play a “scaffold” role in bridging the RC/RO and RE, and this could facilitate targeting endocytosed cholesterol to generate RO membranes. As CVB3 3A and PI4KB bind to ACBD3 (see above), it is possible that ACBD3 could also play a role in bridging RE to the RC.

## 19. Lipid Droplets

Lipid droplets (LDs) are distinct cellular organelles bounded by a single phospholipid monolayer that store neutral lipids to provide energy and also for membrane homeostasis [313]. Lipid droplets establish MCS with many different organelles to facilitate non-vesicular lipid transfer between their membranes [313,314]. Recently, enteroviruses have been shown to establish novel MCS between the RC and LD [86,234,315] and this is thought to provide the lipids for forming RO. In PV-infected cells, 2C and 2BC located to LD and it was suggested that the ability of 2C to self-oligomerize [316] (and/or to interact with 2BC) could allow for formation of a “molecular bridge” that maintains the close spatial association of the RC with LD [315]. PV 3A has been shown to bind to 2C and 2BC and there is also evidence for a functional interaction between 2C and 3A during PV replication [110,111]. Furthermore, co-expression of 3A and 2BC induces formation of membrane structures that are similar to RO in PV-infected cells [236]. Interestingly, the ability of 3AB to interact with only the outer leaflet of membranes (a monotopic interaction) may allow for 3AB (but not 3A) to target LD [317]. Thus, by interacting with other viral proteins it can be speculated that 3AB could also participate in maintaining the close association between the RC and LD.

The study by Laufman et al. [315] also showed that PV 3A and 3AB interact with adipose triglyceride lipase (ATGL) and hormone sensitive lipase (HSL). These are LD-associated enzymes involved in lipid metabolism and it was suggested that by binding to 3A they could promote mobilization of lipid stores for generating RO. Although GBF1 preferentially locates to the Golgi, it also localizes to other organelles such as LD where it facilitates delivery of LD-associated proteins, including ATGL, to the LD membrane [133,318,319,320]. Furthermore, GBF1 has been reported to bind to ATGL and this may contribute to its localization to LD [318]. It has been suggested that the interaction between 3A and ATGL could be mediated by an interaction with GBF1 [321]. Thus, it will be interesting to determine if GBF1 contributes to picornavirus replication when located on LD.

## 20. Conclusions

As described above, many exciting papers have led to picornavirus 3A and 3AB proteins being assigned diverse roles in the viral replication cycle, such as inhibiting protein trafficking (3A), presenting VPg for uridylylation (3AB) and the recruitment of PI4KB to RO (3A/3AB). However, some of these roles have recently been challenged and may be carried out by other viral nsps, and there is much still to learn about the role(s) of 3A/3AB in picornavirus replication.

Perhaps the most intriguing conundrum in enterovirus replication concerns the role of GBF1. Although the precise role of GBF1 remains to be determined, its GEF activity appears to be essential for enterovirus replication, yet the ability of 3A to inhibit its GEF activity is thought to provide a mechanism for some enteroviruses to inhibit protein trafficking. GBF1 is central to the formation of transport vesicles in the early secretory pathway, so inhibition of its GEF activity would be expected to inhibit protein trafficking. However, GBF1 exists as a cytosolic pool and is also recruited to other cellular sites including LD, where it regulates the recruitment of LD-associated proteins [319]. Interestingly, LD are currently thought to be a major source of lipids for formation of RO. It will be interesting to determine if different enterovirus nsps interact with different cellular pools of GBF1, potentially at different stages of replication, with different functional outcomes for its GEF activity. It is conceivable that this could be achieved (at least, in part) by the spatial and temporal regulation of nsps during infection. With this in mind, fully processed 3A is present at a relatively low level during the early phase of infection and may be preferentially recruited to different membrane sites than 3AB due to different membrane interactions (monotopic or bitopic). Furthermore, at least two different labelling patterns (relative to the RC) for 3A have been identified in PV-infected cells [282], which suggest the possibility that a distinct pool of 3A may be spatially segregated from the RC.

The 3A proteins of several picornaviruses bind to ACBD3. An intriguing observation is that the 3A protein of kobuviruses and enteroviruses appear to have evolved the ability to bind to ACBD3 independently with different outcomes for virus replication. The interaction between AiV 3A and ACBD3 redirects PI4KB to RO where it generates PI4P, whereas although enterovirus 3A interactions with ACBD3 appear to redirect PI4KB to the RC it does not induce PI4P synthesis. Unravelling the details of the consequences of the interaction between enterovirus 3A and ACBD3 will undoubtedly herald new lines of investigation and insights into enterovirus replication.

The roles of the 3A proteins produced by other picornaviruses are still poorly understood. For example, HAV 3A binds to ACBD3, but HAV does not require either PI4KA or PI4KB for replication suggesting that the interaction between 3A and ACBD3 provides a novel, but as yet unidentified, function during HAV replication. Similarly, little is known about the role of FMDV 3A in its replication, including the role of its extended C-terminal domain. FMDV 3A does not bind to ACBD3, and FMDV replication is insensitive to BFA (which implies replication does not require GBF1) and does not depend on PI4KA or PI4KB. Thus, it is likely that the 3A proteins of HAV and FMDV will interact with different host proteins (compared to enteroviruses and kobuviruses) and will provide distinct functions during virus replication. As with PV 3A, within FMDV-infected cells, the 3A appears to segregate into at least two distinct populations, which are either associated or not with the RC [248], and it will be interesting to determine if this reflects a separation of independent functional pools of 3A (or 3A and 3AB) at different membrane locations.

A direct role for 3A in evading the cellular innate antiviral responses within infected-cells remains to be clarified and this activity will need to be combined with understanding of the role of viral proteases in antagonizing the cellular antiviral response [185,186,322], and with the global reductions in cellular transcription [82] and translation [323] that occur in picornavirus-infected cells. Each of these processes contributes significantly to the down-regulation of cellular innate immunity. Finally, it is likely that as new picornaviruses are discovered novel roles for 3A/3AB will be uncovered, which will undoubtedly surprise.

## Figures and Tables

**Table 1:** Genome organization and genus specific differences of selected picornaviruses.

Picornavirus General Genome Organization:					
1 x ORF VPg-5’-UTR-IRES -(±L)-1A-1B-1C-1D-2A-2B-2C-3A-3B-3C-3D -3’-UTR-poly(A)					
Genus	IRES			Genus Specific Differences	
Aphthovirus	II	L^pro^		2A^npgp^	3B_1_3B_2_3B_3_
Aalivirus	IV		1AB	2A_1_^npgp^2A_2_^npgp^2A_3_^npgp^2A_4_^npgp^2A_5_2A_6_	
Aquamavirus	IV		1AB	2A_1_^npgp^2A_2_	3B_1_3B_2_
Avihepatovirus	IV		1AB	2A_1_^npgp^2A_2_^NTPase^2A_3_^H-box/NC^	
Cardiovirus	II	L		2A^npgp^	
Enterovirus	I			2A^pro^	
Kobuvirus	V	L	1AB	2A^H-box/NC^	
Limnipivirus	IV		1AB	2A_1_^npgp^2A_2_^npgp^	
Mosavirus	II	L		2A^npgp^	3B_1_3B_2_
Parechovirus	II		1AB	2A_1_^npgp^2A_2_^H-box/NC^	
2 x ORF VPg-5’-UTR-IRES^1^-1A-1B-1C-1D-IGR-IRES^2^-2A-2B-2C-3A-3B-3C-3D -3’-UTR-poly(A)					
Dicipivirus	II^1^ / I ^2^	ORF1 1A-1B-1C-1D		ORF2 2A-2B-2C	ORF2 3A-3B-3C-3D

The genomes of most picornaviruses conform to the general layout of VPg-5’-UTR-Open Reading Frame (ORF)-3’-UTR-poly(A). Internal ribosome entry site (IRES) elements are one of at least five types (I to V). 1A, 1B, 1C and 1D encode the mature capsid proteins. 2A, 2B, 2C, 3A, 3B, 3C and 3D encode the nsps. Genus specific differences are shown. Some picornaviruses encode a Leader protein before 1A (L = Leader protein; L^pro^ = Leader protein has protease activity). 1AB indicates that VP0 (i.e. VP4-VP2) remains un-processed. Some picornaviruses encode more than one 2A protein. The different types of 2A proteins are indicated; 2A^npgp^ = 2A has an NPG/P motif; 2A^NTPase^ = 2A with an NTP-binding motif; 2A^H-box/NC^ = 2A with a H-box/NC motif; 2A^pro^ = 2A has proteinase activity; 2A = 2A lacking a signature motif. When the first copy of 2A has an NPG/P motif it can be expected that the 2A protein forms part of the capsid precursor P1-2A (as shown for FMDV). Some picornaviruses encode more than one 3B (VPg) protein. Dicipiviruses are unusual and have two ORFs that are separated by an intergenic region (IGR) and therefore have two IRES elements.

**Table 2:** Summary of interactions between 3A and 3AB with other nsps.

	Y-2-H [111,112]		M-2-H [110]			M-2-H [109]	
	PV					AiV	
	3A	3AB	3A	3AB		3A	3AB
PV 2A	+	–	ND	ND	AiV 2A	+	ND
PV 2B	+*	+*	+	+	AiV 2B	+	–
PV 2C	+*	+*	+	-	AiV 2C	+	+
PV 2BC	–	–	+	+	AiV 2BC	+	+
PV 3A	+*	+	+	+	AiV 3A	+	+
PV 3B	–	–	ND	ND	AiV 3B	ND	ND
PV 3AB	+	+	+	–	AiV 3AB	+	–
PV 3C	–	–	ND	ND	AiV 3C	–	+
PV 3D	–	+*	ND	ND	AiV 3D	–	–
PV 3CD	–	+*	ND	ND	AiV 3CD	–	+

Summary of interactions between 3A and 3AB with the nsps for poliovirus (PV) and aichivirus (AiV) in the yeast-2-hybrid (Y-2-H) and mammalian-2-hybrid (M-2-H) assay systems [109,110,111,112]. – indicates no interaction observed; + indicates positive interaction was observed in at least one orientation and in one of the studies. Note for the positive interactions, the strength of the interactions was different (see publications for details). ND, not done. Key similarities and differences between the results, which are discussed in the text, are highlighted. Homodimerization interactions for 3A and 3AB are highlighted in yellow (Dark yellow indicates + interaction and light yellow indicates – interaction). 3A:3AB interactions are highlighted in green. For PV and AiV, the 3AB (but not 3A) interacts with 3CD (Blue highlights; dark blue indicates + interaction and light blue indicates – interaction). Only PV 3AB (and not AiV 3AB or PV/AiV 3A) interacted with 3D (highlighted red). Only AiV 3AB (and not PV 3AB or PV/AiV 3A) interacted with 3C (highlighted purple). Only AiV 3AB did not interact with 2B (highlighted orange). It should be noted that the Y-2-H and M-2-H assays rely on interactions between individual picornavirus proteins, as part of a fusion protein, within the nucleus of the cell. This is an atypical environment for the picornavirus proteins and could influence the results. However, for PV, the interaction of 3A with 3A, 2B, 2C, and for 3AB with 2B, 2C, 3D and 3CD (*) have been confirmed using other approaches.

## Data Availability

No new data are reported, only a review of previously published material.
